# Parameter extraction of photovoltaic cell/module models using starfish optimization algorithm with a secant-based objective function modification

**DOI:** 10.1038/s41598-025-34584-1

**Published:** 2026-02-02

**Authors:** Yacine Bouali, Basem Alamri

**Affiliations:** 1 Department of Electrical Engineering, University of Science and Technology Houari Boumediene, P.O. Box 32, El-Alia, Algiers, 16111 Algeria; 2 Department of Electrical Engineering, College of Engineering, Taif University, P.O. Box 11099, Taif, 21944 Saudi Arabia

**Keywords:** Starfish optimization algorithm, Parameter estimation, Single-diode model, Double-diode model, Three-diode model, Secant method, Energy science and technology, Engineering, Mathematics and computing

## Abstract

Accurate identification of photovoltaic (PV) cell and module parameters is essential for reliable electrical modeling, performance assessment, and long-term energy yield prediction. This task is commonly formulated as an optimization problem, where the root mean square error (RMSE) between measured and estimated current-voltage characteristics is minimized. While numerous metaheuristic algorithms have been proposed to solve this problem, most existing studies focus primarily on algorithmic modifications, with limited attention given to enhancing the problem formulation itself. In this work, a recently introduced metaheuristic, the Starfish Optimization Algorithm (SFOA), is employed for PV parameter extraction and systematically evaluated against four contemporary optimization algorithms. In addition, a novel secant-based reformulation of the objective function is proposed to improve the accuracy of the parameter estimation process beyond the conventional RMSE-based approach. The proposed framework is validated on multiple PV models, including the single-diode (SDM), double-diode (DDM), and three-diode (TDM) models for PV cells, as well as the single-diode model of a PV module (PVM). Two widely used benchmark datasets, RTC France and Photowatt-PWP201, are used for experimental verification. The results demonstrate that integrating the secant-based objective function significantly enhances estimation accuracy and robustness across all considered models. In particular, the SFOA-Secant configuration achieves the lowest RMSE values of $7.6579 \times 10^{-4}$ for SDM, $7.4192 \times 10^{-4}$ for DDM, $7.3218 \times 10^{-4}$ for TDM, and $2.0489 \times 10^{-3}$ for PVM, outperforming all competing methods. These findings confirm that reformulating the objective function using the secant method constitutes an effective and complementary strategy for improving PV parameter extraction accuracy.

## Introduction

Solar energy is one of the most important energy sources used in the field of renewable energy^1,2^. In recent years, solar energy has been used in several fields, such as military and medical applications^3,4^. It is increasingly applied in residential and industrial sectors as well, providing a sustainable alternative to fossil fuels^5,6^. Solar panels are now widely installed on rooftops, powering homes, schools, and businesses with clean electricity^7^. Moreover, the development of grid-connected photovoltaic (PV) systems has significantly enhanced the efficiency and reliability of solar energy utilization^8^, enabling excess energy to be integrated into the grid and guaranteeing a consistent power supply, even during periods of limited solar generation.

The efficiency of PV cell technology has steadily improved through advancements in cell design, material selection, and fabrication techniques^9–11^. To accurately analyze and predict the performance of PV cells under various operating conditions, researchers commonly employ mathematical modeling^12^. In the literature, three primary models are widely used for simulation: the single-diode model (SDM), the double-diode model (DDM), and the three-diode model (TDM)^13^. However, these models contain different unknown parameters that must be identified before they can be effectively used for simulation and practical applications^14^.

In the literature, the identification of PV parameters is commonly formulated as an optimization problem, where the root mean square error (RMSE) is typically employed as the objective function to minimize the discrepancy between the experimental data and the estimated data obtained from the extracted parameters.

In^15^, the authors proposed the mother tree optimization with climate change (MTO-CL) algorithm to enhance parameter estimation in the three-diode PV model. The method achieved greater accuracy and robustness than seven other algorithms by adding elimination and distortion phases based on climate dynamics. This resulted in remarkably low RMSE and power errors across different modules. But this increase in accuracy comes with a longer computational time, which makes it less advantageous for real-time applications. In^16^, a modified electric eel foraging optimization (MEEFO) algorithm incorporating fractional-order calculus, fitness distance balance, and quasi-opposition-based learning to improve PV parameter estimation. The method demonstrated superior exploration and exploitation capabilities, avoiding premature convergence and achieving the lowest RMSE values across single, double, and triple diode models under varying meteorological conditions. However, the added strategies increase algorithmic complexity, which may limit its adaptability for real-time applications. In^17^, the research introduced the Levy flight and mutation-enhanced artificial rabbit optimization (LMARO) algorithm for PV parameter extraction. By combining swarm-elite learning, Levy flight, and mutation strategies, LMARO improved global exploration, population diversity, and convergence speed, achieving competitive RMSE values across single-diode, double-diode, and PV module models. While the method demonstrated strong accuracy and stability compared to several advanced algorithms, its adaptability to broader optimization problems still requires further improvement.

In^18^, the authors presented the bio-dynamics grasshopper optimization algorithm (BDGOA) aimed at improving photovoltaic parameter identification. The method enhanced convergence speed, exploration, and robustness relative to GOA, effectively estimating parameters of various commercial PV modules across different conditions; however, temperature-dependent variations in specific parameters persisted. In^19^, the paper suggested the enhanced prairie dog optimizer (En-PDO), which integrates random learning and logarithmic spiral search to improve PV parameter identification. Compared with the original PDO and eighteen recent algorithms, En-PDO consistently achieved lower RMSE values across single-, double-, and triple-diode models as well as PV module models under diverse conditions. While the method proved robust and accurate, future improvements are needed to extend its adaptability for dynamic environments and real-time applications. In^20^, the work proposed a hybrid multi-population gorilla troops optimizer and beluga whale optimization (HGTO-BWO) to improve PV parameter extraction. By combining multi-population strategies with exploration-exploitation mechanisms such as Levy flight and synchronized motion, the method achieved the lowest RMSE values across double- and triple-diode models for various PV cells and modules under different operating conditions. Despite its high accuracy and robustness, the approach is computationally demanding and complex to implement, limiting its practicality without further simplification.

In^21^, a hybrid kepler optimization algorithm (HKOA) was introduced by enhancing KOA with ranking-based update and exploitation-improvement mechanisms. These additions strengthen exploration to avoid local optima and improve exploitation for faster convergence. HKOA outperformed several recent algorithms on the RTC France cell and multiple PV modules, showing strong accuracy and stability. However, its reliance on extra control parameters and relatively high computational cost remain notable limitations. In^22^, a hybrid flower grey differential (HFGD) algorithm combining flower pollination algorithm (FPA), grey wolf optimizer (GWO), and differential evolution (DE) algorithm; was proposed to improve PV parameter estimation. With added Newton-Raphson refinement, HFGD achieved the lowest RMSE and strong robustness across several PV models. Its main drawback is the increased algorithmic complexity arising from multi-hybrid integration. In^23^, a kangaroo escape optimization (KEO) algorithm was proposed by modeling kangaroos’ escape behavior through chaotic energy adaptation, zigzag exploration, long-jump motions, and decoy-based exploitation. KEO achieved high accuracy and robustness across SDM, DDM, and TDM on the RTC France and Photowatt-PWP201 datasets, outperforming several recent optimizers. Its improved exploration-exploitation balance makes it effective for complex nonlinear PV models, though its multi-stage update strategy adds complexity that may affect computational efficiency in real-time scenarios.

In^24^, the authors introduced reconfigured single- and double-diode models (Reconfig-SDM and Reconfig-DDM) by adding a small series resistance to the diode branches to better capture PV nonlinearities. Using the squirrel search algorithm for parameter extraction, the proposed models achieved notably lower RMSE than classical SDM/DDM on RTC France and CS6P-220P modules. However, the improved accuracy comes at the cost of increased model complexity, requiring more parameters and computational effort during estimation. In^25^, an enhanced differential evolution (EDE) algorithm was introduced using stage-specific mutation and crossover strategies to improve exploration and exploitation during PV parameter estimation. EDE achieved the lowest RMSE across SDM, DDM, and TDM for multiple cells and modules, showing strong accuracy and convergence reliability. Its main limitation is the increased algorithmic complexity from adaptive control parameters, which may hinder real-time deployment. In^26^, the improved sinh cosh optimizer (ISCHO) enhances SCHO with trigonometric operators to strengthen exploitation and avoid local optima. It achieves low RMSE and reliable performance across multiple PV models and modules, outperforming several recent methods. However, the added operators increase computational complexity, limiting its application in real-time settings.

In recent years, researchers have employed various metaheuristic optimization algorithms to tackle the challenge of estimating PV parameters. These algorithms include: a hybrid optimization technique combining the analytical Newton-Raphson-based optimization (Ana-NRBO) algorithm with an analytical initialization method^27^, wild horse optimizer^28^, shuffled puma optimizer^29^, frilled lizard optimization^30^, snake optimization with sine-cosine algorithm^31^, PID-based search algorithm (PSA)^32^, generalized normal distribution optimization based on neighborhood search strategies (NSGNDO)^33^, pelican optimization algorithm^34^, drone squadron optimization^35^, lungs performance-based optimization (LPO) algorithm^36^, gold rush optimizer^37^, archimedes optimization algorithm^38^, improved simultaneous heat transfer search^39^, butterfly optimization algorithm with chaos learning strategy^40^, northern goshawk optimization algorithm^41^, tree seed algorithm^42^, nutcracker optimization algorithm^43^, landscape-aware particle swarm optimization (LaPSO)^44^, adaptive slime mould algorithm (ASMA)^45^, weighted mean of vectors (INFO)^46^, chaotic-gradient-based optimizer^47^, modified elephant herding optimization^48^, improved bonobo optimizer^49^, gradient-based optimizer^50^, and coyote optimization algorithm^51^.

Although numerous metaheuristic algorithms have been proposed for PV parameter extraction, most existing studies concentrate primarily on algorithmic improvements while relying on the same conventional problem formulation based on the RMSE objective function. This narrow focus overlooks the fact that the accuracy and convergence behavior of any optimizer are strongly influenced not only by the search mechanism but also by the mathematical structure of the objective function itself. As a result, even recently introduced high-performance optimizer may still exhibit slow convergence, stagnation, or sensitivity to initial conditions when the underlying formulation remains unchanged.

A limited number of works have attempted to modify the problem formulation directly^13,52^. For example, in^13^ proposed the Flood Algorithm (FLA) along with a Newton–Raphson based objective function. While their modified formulation enhanced accuracy, it introduced substantial computational overhead due to derivative evaluations, making it unsuitable for real-time or embedded PV applications. This highlights two key limitations in the current literature: derivative-based objective reformulations increase computational cost and sensitivity to numerical instabilities, and metaheuristic algorithms enhancements alone cannot fully address the inherent nonlinearity of PV models when the traditional RMSE formulation remains unchanged.

Motivated by these gaps, this work introduces a secant-based objective function modification as a derivative-free reformulation of the PV parameter extraction problem. Unlike the traditional formulation of the objective function, the secant mechanism approximates the estimated current used in the RMSE formula without requiring explicit derivatives, thereby maintaining numerical stability. This modification enabling faster convergence toward high-accuracy solutions.

In parallel, and in accordance with the No Free Lunch (NFL) theorem, which states that no single optimizer performs best across all problem classes^53^. A recently developed metaheuristic, the starfish optimization algorithm (SFOA), is employed. SFOA offers a well-balanced exploration-exploitation structure inspired by starfish sensory-driven movement and regeneration behavior^54^. Its multi-arm search mechanism enables effective coverage of high-dimensional spaces, while its regeneration phase helps escape local minima; an essential capability for the highly multimodal PV parameter extraction problem. Compared to traditional nature-inspired metaheuristic algorithms, SFOA does not rely on delicate control parameters, making it robust and easier to implement.

This paper investigates the secant method to enhance problem formulation, aiming to achieve a small RMSE value compared to the traditional formulation of the PV parameters extraction objective function. The key contributions of this paper can be summarized as follows:

- Application of the starfish optimization algorithm: this recently introduced metaheuristic is employed for accurate extraction of parameters in PV cell and module models.
- Comparative performance analysis: the results obtained using SFOA are rigorously compared with those from four other state-of-the-art optimization algorithms, educational competition optimizer (ECO)^55^, hippopotamus optimization algorithm (HO)^56^, osprey optimization algorithm (OOA)^57^, and zebra optimization algorithm (ZOA)^58^.
- Objective function enhancement: the objective function is reformulated using the secant method, and its performance is evaluated against the conventional objective functions commonly reported in the literature.
- The combination of SFOA with the secant-based formulation achieves superior RMSE performance across SDM, DDM, TDM, and PV module models, confirming the effectiveness of both the new problem formulation and the optimizer.

This paper is organized as follows. The section on PV Models and Problem Formulation outlines the photovoltaic cell and module models and formulates the parameter extraction problem, including the secant-based objective function. The Starfish Optimization Algorithm section presents a detailed description of the proposed optimization method. The Experimental Setup section describes the benchmark datasets, error metrics, and comparative optimization algorithms. The Results and Discussion section presents and analyzes the obtained results. Finally, the Conclusion section summarizes the main findings and highlights potential directions for future research.

## PV models and problem formulation

### PV cell/module models

Accurate design of PV cells and modules requires both a precise mathematical model and an efficient metaheuristic algorithm to estimate the model’s unknown parameters. In the literature, the single-diode, double-diode, and three-diode models are widely used for PV cells, while the single-diode model (SDM) is typically employed for PV modules. These models are described in this section.

**Fig. 1:**
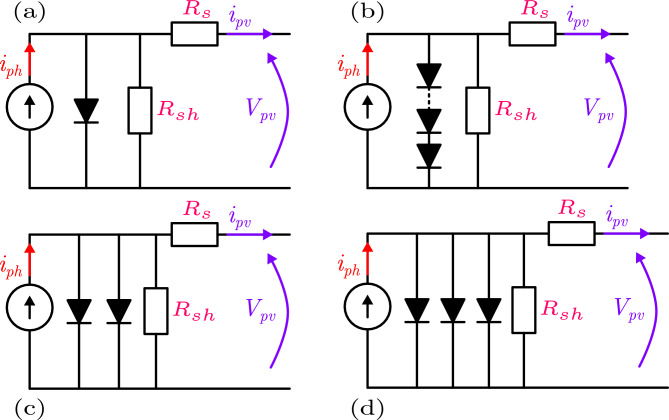
Equivalent circuit of (**a**) SDM, (**b**) PVM, (**c**) DDM, and (**d**) TDM.

The electrical equivalent circuit of the SDM is shown in Fig. 1a. It consists of a constant current source, a diode, a shunt (parallel) resistance, and a series resistance. The diode models the p-n junction of the solar cell, representing the diffusion current due to carrier transport across the junction. By applying Kirchhoff’s current law, the output current of the SDM can be expressed as^13^:

1 $$\begin{aligned} I_{pv} = I_{ph} - I_{sd} \left[ exp \left( \frac{q(V_{pv}+I_{pv}R_{s})}{nkT} \right) - 1 \right] - \frac{V_{pv} + I_{pv} R_{s}}{R_{sh}} \end{aligned}$$

In this equation, $I_{pv}$ is the output current of the photovoltaic (PV) cell, and $I_{ph}$ is the photocurrent generated by the incident light. The term $I_{sd}$ denotes the reverse saturation current of the diode. The constants *k* and *q* represent the Boltzmann constant and the elementary charge, with values 1.38064852 $\times 10^{-23}$ J/K and 1.6021764 $\times 10^{-19}$ C, respectively.

The parameter *n* is the diode ideality factor, and *T* is the temperature of the PV cell in Kelvin. $V_{pv}$ denotes the output voltage of the PV cell. The terms $R_s$ and $R_{sh}$ represent the series resistance and the shunt resistance of the PV cell, respectively.

PV modules typically consist of $N_s$ solar cells connected in series to meet the desired power output, as shown in Fig. 1b. The output current of the PV module model (PVM) is given by^13^:

2 $$\begin{aligned} I_{pv} = I_{ph} - I_{sd} \left[ exp \left( \frac{q(V_{pv}+N_{s}I_{pv}R_{s})}{nkTN_{s}} \right) - 1 \right] - \frac{V_{pv} + N_{s}I_{pv} R_{s}}{N_{s}R_{sh}} \end{aligned}$$

After excluding the known parameters, both the SDM and PVM have five unknown parameters that require estimation, defined as $X = \left[ I_{ph}, I_{sd}, R_{sh}, R_{s}, n \right]$.

The Double-Diode Model (DDM), shown in Fig. 1c, contains the same elements as the SDM but includes a second diode connected in parallel with the first. The second diode models the recombination current in the depletion (space-charge) region, while the first diode represents the diffusion current as in the SDM. This model provides better accuracy under low-irradiance conditions or for high-quality cells where recombination effects in the depletion region are significant. The output current of the DDM is^18^:

3 $$\begin{aligned} \begin{aligned} I_{pv} = I_{ph}&- I_{sd1} \left[ exp \left( \frac{q(V_{pv}+I_{pv}R_{s})}{n_1kT} \right) - 1 \right] \\&- I_{sd2} \left[ exp \left( \frac{q(V_{pv}+I_{pv}R_{s})}{n_2kT} \right) - 1 \right] \\&- \frac{V_{pv} + I_{pv} R_{s}}{R_{sh}} \end{aligned} \end{aligned}$$

In this model, $I_{sd1}$ and $I_{sd2}$ denote the saturation currents due to diffusion and recombination, respectively, while $n_1$ and $n_2$ represent the corresponding ideality factors of the diodes.

By excluding the known parameters, the DDM is characterized by seven unknown parameters that need to be estimated: $X = \left[ I_{ph}, I_{sd1}, I_{sd2}, R_{sh}, R_{s}, n_1, n_2 \right]$.

The Three-Diode Model (TDM), shown in Fig. 1d, extends the DDM by adding a third diode in parallel. This additional diode captures further nonlinear effects, such as junction breakdown, defect-related leakage, or recombination at grain boundaries in polycrystalline cells. The TDM is particularly useful for modeling cells under low-irradiance conditions. The output current of the TDM is given by^46^:

4 $$\begin{aligned} \begin{aligned} I_{pv} = I_{ph}&- I_{sd1} \left[ exp \left( \frac{q(V_{pv}+I_{pv}R_{s})}{n_1kT} \right) - 1 \right] \\&- I_{sd2} \left[ exp \left( \frac{q(V_{pv}+I_{pv}R_{s})}{n_2kT} \right) - 1 \right] \\&- I_{sd3} \left[ exp \left( \frac{q(V_{pv}+I_{pv}R_{s})}{n_3kT} \right) - 1 \right] \\&- \frac{V_{pv} + I_{pv} R_{s}}{R_{sh}} \end{aligned} \end{aligned}$$

In this model, $I_{sd3}$ represents the additional saturation current associated with a third recombination mechanism, often included to capture more complex carrier dynamics and improve the accuracy of the PV cell modeling under various operating conditions.

After excluding the known parameters, the triple-diode model (TDM) comprises nine unknown parameters that must be estimated: $X = \left[ I_{ph}, I_{sd1}, I_{sd2}, I_{sd3}, R_{sh}, R_{s}, n_1, n_2, n_3 \right]$.

### Secant-based objective function

Parameter identification of a PV cell/module is treated as an optimization problem, aiming to determine the set of parameters that most closely matches the experimental I–V curve of the PV cell/module. Modeling a PV cell/module involves the precise determination of the parameters in Eqs. (1), (2), (3), and (4). For that, an objective function is defined as follows:

5 $$\begin{aligned} RMSE(X) = \sqrt{\frac{1}{N_{m}} \sum _{i=1}^{N_{m}} f_M(V_{pv_{i}},I_{pv_{i}},X)^2} \end{aligned}$$

where *X* is the vector of parameters to be extracted for each model, $N_{m}$ is the number of experimental data points, and *M* is the model type. The function $f_M$ for each model is defined as follows:

6 $$\begin{aligned} \begin{aligned} f_{SDM}(V_{pv},I_{pv},X) = I_{ph}&- I_{sd} \left[ exp \left( \frac{q(V_{pv}+I_{pv}R_{s})}{nkT} \right) - 1 \right] \\&- \frac{V_{pv} + I_{pv} R_{s}}{R_{sh}} - I_{pv} \end{aligned} \end{aligned}$$

7 $$\begin{aligned} \begin{aligned} f_{DDM}(V_{pv},I_{pv},X) = I_{ph}&- I_{sd1} \left[ exp \left( \frac{q(V_{pv}+I_{pv}R_{s})}{n_1kT} \right) - 1 \right] \\&- I_{sd2} \left[ exp \left( \frac{q(V_{pv}+I_{pv}R_{s})}{n_2kT} \right) - 1 \right] \\&- \frac{V_{pv} + I_{pv} R_{s}}{R_{sh}} - I_{pv} \end{aligned} \end{aligned}$$

8 $$\begin{aligned} \begin{aligned} f_{TDM}(V_{pv},I_{pv},X) = I_{ph}&- I_{sd1} \left[ exp \left( \frac{q(V_{pv}+I_{pv}R_{s})}{n_1kT} \right) - 1 \right] \\&- I_{sd2} \left[ exp \left( \frac{q(V_{pv}+I_{pv}R_{s})}{n_2kT} \right) - 1 \right] \\&- I_{sd3} \left[ exp \left( \frac{q(V_{pv}+I_{pv}R_{s})}{n_3kT} \right) - 1 \right] \\&- \frac{V_{pv} + I_{pv} R_{s}}{R_{sh}} - I_{pv} \end{aligned} \end{aligned}$$

9 $$\begin{aligned} \begin{aligned} f_{PVM}(V_{pv},I_{pv},X) = I_{ph}&- I_{sd} \left[ exp \left( \frac{q(V_{pv}+N_{s}I_{pv}R_{s})}{nkTN_{s}} \right) - 1 \right] \\ &- \frac{V_{pv} + N_{s}I_{pv} R_{s}}{N_{s}R_{sh}} - I_{pv} \end{aligned} \end{aligned}$$

In this approach, $V_{pv}$ and $I_{pv}$ are replaced by the measured data to compute the error between the measured current $I_{m}$ and the estimated current $I_{e}$ based on the candidate parameter set *X*.

For all PV models considered in this study (SDM, PVM, DDM, and TDM), the current–voltage relationship can be expressed in the implicit nonlinear form $f_M(V_{pv}, I_{pv}, X) = 0$, Due to the coexistence of linear and exponential current terms, this equation does not admit a closed-form solution for $I_{pv}$. The conventional objective function minimizes the algebraic residual of equation (5). This approach does not enforce physical consistency and results in a rugged fitness landscape due to the strong nonlinearity of $f_M$.

In the literature, this approach is the most common in papers on parameter extraction of PV cells/modules. However, in this paper, a new approach is proposed based on finding the estimated current $I_{e}$ using the secant method, then calculating the RMSE.

To overcome the limitation of conventional approach, the estimated current $I_{e}$ is obtained by explicitly solving $f_M(V_{pv}, I_{pv}, X) = 0$. for each voltage point using the secant method. The objective function is then defined as:

10 $$\begin{aligned} RMSE = \sqrt{\frac{1}{N_{m}} \sum _{i=1}^{N_{m}} (I_{e_{i}} - I_{m_{i}})^2} \end{aligned}$$

which ensures that the PV model equation is satisfied for every candidate solution.

The secant method is adopted due to its derivative-free nature and superlinear convergence, making it numerically robust when embedded within population-based metaheuristic optimization.

The *secant method* is an iterative, derivative-free algorithm for finding roots of nonlinear equations *g*(*x*). Lets consider

$$g(x) = 0$$

It starts with two initial approximations, $x_0$ and $x_1$, and at each step computes a new approximation $x_{n+1}$ as the x-intercept of the secant line passing through the points

$$\big ( x_{n-1}, g(x_{n-1}) \big ) \quad \text {and} \quad \big ( x_n, g(x_n) \big ).$$

The iteration formula is:

11 $$\begin{aligned} x_{n+1} = x_n - g(x_n) \cdot \frac{x_n - x_{n-1}}{g(x_n) - g(x_{n-1})}. \end{aligned}$$

In contrast to Newton’s method, which requires the exact derivative $g'(x)$, the secant method approximates it by a finite difference:

$$g'(x_n) \approx \frac{g(x_n) - g(x_{n-1})}{x_n - x_{n-1}}.$$

This avoids derivative evaluations while still achieving superlinear convergence under suitable smoothness conditions.

**Fig. 2:**
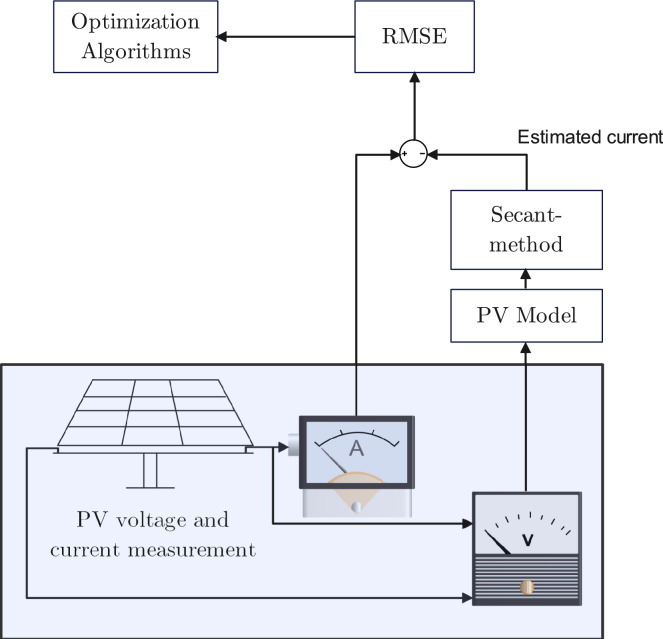
The process of objective function formulation based on Secant-method.

For each model, $f_{M}$ is replaced by the difference between the measured current $I_{m}$ and the estimated current $I_{e}$ as in (10), where $I_{e}$ is the root of $f_{M}$, found using the secant method. The whole process of this approach is shown in Fig. 2.

## Starfish optimization algorithm

The Starfish Optimization Algorithm (SFOA) was proposed in^54^. The inspiration behind the SFOA comes from the unique biological and behavioral characteristics of starfish. Specifically, SFOA draws from starfish’s abilities to explore their surroundings using five arms (each equipped with light-sensitive eyes), their distinctive preying method of everting their stomachs to digest food externally, and their remarkable capacity for regeneration.

### Mathematical model of SFOA

#### Initialization

During the initialization phase of SFOA, starfish positions are randomly generated within the bounds of the design variables and represented in matrix form:

12 $$\begin{aligned} X=\begin{bmatrix} X_{11} & X_{12} & \dots & X_{1D}\\ X_{21} & X_{22} & \dots & X_{2D}\\ \vdots & \vdots & \ddots & \vdots \\ X_{N1} & X_{N2} & \dots & X_{ND} \end{bmatrix}_{N \times D} \end{aligned}$$

where, *X* denote the matrix representing the positions of the starfish, with dimensions $N \times D$, where *N* is the population size and *D* is the number of design variables. In the initialization phase, the position of each starfish is computed according to the following equation:

13 $$\begin{aligned} X_{ij} = L_j + r (U_j - L_j), \quad i=1,2,\dots ,N, \quad j=1,2,\dots ,D \end{aligned}$$

here, $X_{ij}$ denotes the position of the *i*th starfish in the *j*th dimension, *r* is a uniformly distributed random number in the range (0, 1), and $U_j$ and $L_j$ represent the upper and lower bounds of the design variable in the *j*th dimension, respectively. Once the initial position matrix is generated, the fitness values of all starfish can be computed using the objective function and stored in a vector:

14 $$\begin{aligned} F = \begin{bmatrix} F(X_1)\\ F(X_2)\\ \vdots \\ F(X_N) \end{bmatrix}_{N \times 1} \end{aligned}$$

here, *F* is a matrix of size $N \times 1$ used to store and update the fitness values. After initialization, SFOA enters its main loop, beginning the exploration and exploitation phases.

#### Exploration phase

The exploration phase of the SFOA algorithm simulates the starfish’s search capability, inspired by its five arms, each ending with an eye to aid in environmental sensing. In the exploration phase of SFOA, a novel search strategy is introduced, which combines a five-dimensional search pattern for cases where $D>5$, and a unidimensional search pattern when $D\le 5$, depending on the nature of the optimization problem. The dimensional threshold is inspired by the biological structure of starfish, which possess five arms (or eyes), serving as a natural basis for this design.

If the dimension of the optimization problem exceeds 5 ($D>5$), the search space becomes significantly large, requiring the starfish to utilize all five arms to explore its surroundings effectively. Moreover, each arm relies on knowledge of the best position found by the search agents to guide its movement. Based on this, a mathematical model is developed to represent this phase as follows:

15 $$\begin{aligned} Y_{i,p}^{T} = {\left\{ \begin{array}{ll} X_{i,p}^{T} + a_1 \left( X_{best,p}^{T} - X_{i,p}^{T}\right) \cos \theta , \quad r \le 0.5\\ X_{i,p}^{T} - a_1 \left( X_{best,p}^{T} - X_{i,p}^{T}\right) \sin \theta , \quad r > 0.5 \end{array}\right. } \end{aligned}$$

here, $Y_{i,p}^{T}$ and $X_{i,p}^{T}$ represent the updated and current positions of the *i*-th starfish in the *p*-th dimension, respectively. $X_{best,p}^{T}$ denotes the *p*-th dimension of the current best position. The vector *p* consists of five randomly selected dimensions from the total *D* dimensions, and *r* is a random number in the range (0, 1). The parameters $a_1$ and $\theta$ are computed as follows:

16 $$\begin{aligned} a_1 = (2r-1)\pi \end{aligned}$$

17 $$\begin{aligned} \theta = \frac{\pi }{2} \frac{T}{T_{max}},\quad \theta \in \left[ 0, \pi /2\right] \end{aligned}$$

here, *T* denotes the current iteration, while $T_{max}$ is the maximum number of iterations. The sine and cosine components reflect the equal probability of a starfish arm twisting left or right in its attempt to approach food.

For optimization problems where $D>5$, the five-dimensional search pattern from (15) is applied to update only five dimensions of each position. This approach enhances search capability and improves efficiency compared to a full vector-based search pattern.

If an updated position falls outside the boundaries of the design variables, the starfish’s arms tend to remain at their previous positions rather than adopting the invalid update. This behavior can be mathematically expressed as follows:

18 $$\begin{aligned} X_{i,p}^{T+1} = {\left\{ \begin{array}{ll} Y_{i,p}^{T} \quad & L_{b,p} \le Y_{i,p}^{T} \le U_{b,p}\\ X_{i,p}^{T} \quad & \text {otherwise} \end{array}\right. } \end{aligned}$$

where *p* denotes the updated dimension, $L_{b,p}$ and $U_{b,p}$ represent the bounds of design variables, respectively.

If the dimension of the optimization problem is less than or equal to 5 ($D\le 5$), the exploration phase adopts a unidimensional search pattern to update positions. In this case, only one arm of the starfish moves to search for a food source, guided by the positional information of other starfish. The updated position in this scenario is defined as follows:

19 $$\begin{aligned} Y_{i,q}^{T} = E_t X_{i,p}^{T} + A_1 \left( X_{k_1,p}^{T} - X_{i,p}^{T} \right) + A_2 \left( X_{k_2,p}^{T} - X_{i,p}^{T} \right) \end{aligned}$$

here, $X_{k_1,p}^{T}$ and $X_{k_2,p}^{T}$ represent the *p*-th dimensional positions of two randomly selected starfish. $A_1$ and $A_2$ are random numbers within the range $(-1, 1)$, and *p* is a randomly selected dimension from the *D* total dimensions. $E_t$ denotes the energy of the starfish, calculated as follows:

20 $$\begin{aligned} E_t = \frac{T_{max} - T}{T_{max}} \cos \theta \end{aligned}$$

Similar to the previous update rule, if the newly obtained position of a starfish falls outside the boundary, the starfish will remain at its previous position rather than adopting the updated one.

#### Exploitation phase

In SFOA, the exploitation phase focuses on searching for global solutions through two distinct updating strategies: preying and regeneration.

The parallel two-directional search strategy is used to model the preying phase of starfish; in this strategy, the SOFA needs to utilize the information from other starfish and the current best position of the population. First, five distances are computed between the best-known position and those of other starfish. Then, two of these distances are randomly selected to guide the position update of each starfish using a parallel two-directional search strategy. These distances are calculated as follows:

21 $$\begin{aligned} d_m = \left( X_{best}^{T} - X_{m_{p}}^{T}\right) , \quad m=1,\dots ,5 \end{aligned}$$

here, $d_m$ represents the five distances between the global best starfish and five other selected starfish, while $m_{p}$ denotes five randomly chosen starfish. Based on this, the position update rule during the starfish’s preying behavior is defined as follows:

22 $$\begin{aligned} Y_{i}^{T} = X_{i}^{T} + r_1 d_{m_{1}} + r_2 d_{m_{2}} \end{aligned}$$

where, $r_1$ and $r_2$ are random numbers in the range (0, 1), while $d_{m_{1}}$ and $d_{m_{2}}$ are randomly selected values from the set $d_m$.

In addition, starfish are vulnerable to predators during predation due to their slow movement. When threatened, a starfish may escape by shedding an arm, a defensive mechanism used to evade capture.

Consequently, the regeneration phase in SFOA is applied exclusively to the last starfish in the population ($i=N$). Since regeneration in nature takes several months, this phase is modeled with a very slow movement speed. Accordingly, the position update rule for the regeneration phase is defined as follows:

23 $$\begin{aligned} Y_{i}^{T} = exp\left( -T\times N/T_{max} \right) X_{i}^{T} \end{aligned}$$

If the position obtained from (22) or (23) exceeds the boundaries of the design variables, it is adjusted as follows:

24 $$\begin{aligned} X_{i}^{T+1} = {\left\{ \begin{array}{ll} Y_{i}^{T} \quad L_b \le & Y_{i}^{T} \le U_b\\ L_b \quad & Y_{i}^{T} \le L_b\\ U_b \quad & Y_{i}^{T} > U_b \end{array}\right. } \end{aligned}$$

### The procedure and complexity of SFOA

**Fig. 3:**
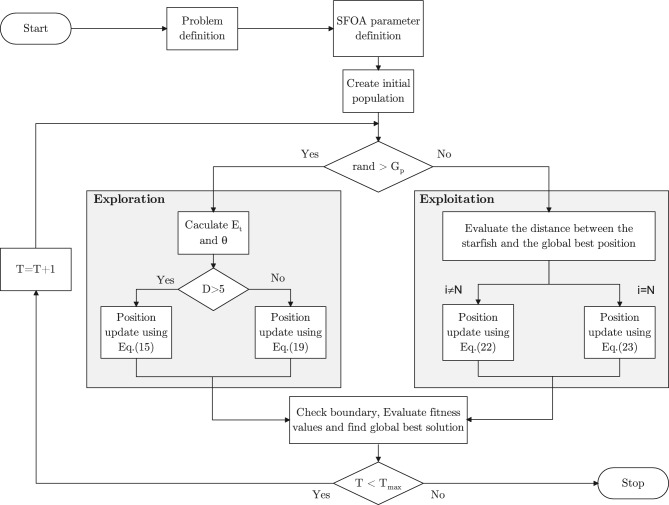
The flowchart of SFOA.

The detailed procedure of SFOA is illustrated in Fig. 3, which presents the algorithm’s flowchart.

The SFOA begins with the initialization phase, where algorithm parameters and problem-specific information are provided. The population is then randomly generated within the design variable boundaries using (12), followed by the evaluation of fitness values. After initialization, SFOA proceeds into the main optimization loop.

In the main loop, the decision to proceed with either the exploration or exploitation phase is based on comparing a random number in the range (0, 1) with the algorithmic parameter $G_p$, which is set to 0.5 in SFOA based on the original paper^54^. When the maximum iteration criterion is met, the main loop terminates, and the final global solution is returned.

At the initialization phase, the computational complexity of SFOA is $O(N \times D)$. During the exploration phase, a hybrid search pattern is employed depending on the dimensionality: for $D>5$, the complexity is $O( 1/2 \times T_{max} \times N \times 5)$, and for $D \le 5$, it is $O( 1/2 \times T_{max} \times N \times 1)$. In the exploitation phase, the complexity is given by $O( 1/2 \times T_{max} \times N \times D)$.

For an optimization problem where $D>5$, the total computational complexity of SFOA is calculated as follows:

25 $$\begin{aligned} O(\text {SOFA}) \simeq O \left( N \times T_{max} \times D \times \left[ \frac{1}{2} + \frac{5}{2D}\right] \right) \end{aligned}$$

For the case where $D\le 5$, the total computational complexity of SFOA is calculated as follows:

26 $$\begin{aligned} O(\text {SOFA}) \simeq O \left( N \times T_{max} \times D \times \left[ \frac{1}{2} + \frac{1}{2D}\right] \right) \end{aligned}$$

To determine the computational complexity of the proposed method, the time complexity of SFOA is combined with that of the Secant-based objective function, denoted as $O(\text {Secant-OF})$. Accordingly, the overall time complexity of the proposed method can be expressed as:

27 $$\begin{aligned} O(\text {Proposed Method}) = O(\text {SFOA}) + O(\text {Secant-OF}). \end{aligned}$$

The computational complexity of the Secant-based objective function is approximated by

28 $$\begin{aligned} O(\text {Secant-OF}) \simeq O \left( N_m \times L_s \right) , \end{aligned}$$

where $N_m$ represents the size of the dataset used for each model, and $L_s$ denotes the number of iterations required by the Secant method. In the other hand, the space complexity of the proposed method is identical to that of SFOA and is approximated as $O \left( N \times D \right)$.

## Experimental setup

### Benchmark data

The benchmark data used for the PV cell are from the RTC France monocrystalline silicon cell, which has a diameter of 57 mm and consists of a single cell. Its experimental I–V curve was obtained under an incident irradiance of 1000 W/m^2^ at an operating temperature of 33 °C, and is characterized by 26 pairs of current–voltage data points. The PV module data are from the Photowatt-PWP201 polycrystalline module, which is composed of 36 cells connected in series. Its experimental I–V curve was measured under an irradiance of 1000 W/m^2^ at an operating temperature of 45 °C, and is characterized by 25 pairs of current–voltage data points. The experimental data are shown in Table 1.

**Table 1:** The experimental voltage and current for RTC France solar cell and Photowatt-PWP201 module.

Data	RTC France^13,18^		Photowatt-PWP201^13,18^	
	$V_{m}$	$I_{m}$	$V_{m}$	$I_{m}$
1	− 0.2057	0.7640	0.1248	1.0315
2	− 0.1291	0.7620	1.8093	1.0300
3	− 0.0588	0.7605	3.3511	1.0260
4	0.0057	0.7605	4.7622	1.0220
5	0.0646	0.7600	6.0538	1.0180
6	0.1185	0.7590	7.2364	1.0155
7	0.1678	0.7570	8.3189	1.0140
8	0.2132	0.7570	9.3097	1.0100
9	0.2545	0.7555	10.2163	1.0035
10	0.2924	0.7540	11.0449	0.9880
11	0.3269	0.7505	11.8018	0.9630
12	0.3585	0.7465	12.4929	0.9255
13	0.3873	0.7385	13.1231	0.8725
14	0.4137	0.7280	13.6983	0.8075
15	0.4373	0.7065	14.2221	0.7265
16	0.4590	0.6755	14.6995	0.6345
17	0.4784	0.6320	15.1346	0.5345
18	0.4960	0.5730	15.5311	0.4275
19	0.5119	0.4990	15.8929	0.3185
20	0.5265	0.4130	16.2229	0.2085
21	0.5398	0.3165	16.5241	0.1010
22	0.5521	0.2120	16.7987	− 0.0080
23	0.5633	0.1035	17.0499	− 0.1110
24	0.5736	− 0.0100	17.2793	− 0.2090
25	0.5833	− 0.1230	17.4885	− 0.3030
26	0.5900	− 0.2100	–	–

Table 2 lists the parameter ranges for each PV cell/module model, which are identical to those reported in previous studies^13,18^.

**Table 2:** Parameters boundaries of RTC France PV cell and PhotoWatt PWP-201 PV module.

Parameter	RTC France^13,18^		PhotoWatt-PWP201^13,18^	
	LB	UB	LB	UB
$I_{ph}$ (*A*)	0	1	0	2
$I_{sd}$ ($\mu A$)	0	1	0	50
$I_{sd1},I_{sd2},I_{sd3}$ ($\mu A$)	0	1	–	–
*n*	1	2	1	50
$n_{1},n_{2}$	1	2	–	–
$n_{3}$	2	5	–	–
$R_{sh}$ ($\Omega$)	0	100	0	1000
$R_{s}$ ($\Omega$)	0	0.5	0	2

### Error metrics

To evaluate the accuracy of the estimated current values in comparison to the measured data, the following error metrics are used:

- *Absolute error (AE)* represents the total magnitude of the deviation between the measured current values $I_{m,i}$ and the estimated current values $I_{e,i}$, summed over all data points.
  29 $$\begin{aligned} AE = \sum _{i=1}^{N_{m}} |I_{m,i} - I_{e,i} |\end{aligned}$$
- *Mean absolute error (MAE)* measures the average magnitude of the absolute differences between measured and estimated currents, providing a general indication of prediction accuracy.
  30 $$\begin{aligned} MAE = \frac{1}{N_{m}} \sum _{i=1}^{N_{m}} |I_{m,i} - I_{e,i} |\end{aligned}$$
- *Maximum absolute error (MaxAE)* is the maximum absolute difference between the measured and estimated currents over all data points. It represents the worst-case error.
  31 $$\begin{aligned} MaxAE = \max _{i=1,\dots ,N_{m}} |I_{m,i} - I_{e,i} |\end{aligned}$$
- *Median bias error (MBE)* represents the average signed difference between measured and estimated currents. A positive MBE indicates underestimation by the model, while a negative value indicates overestimation.
  32 $$\begin{aligned} MBE = \sum _{i=1}^{N_{m}} \frac{(I_{m,i} - I_{e,i})}{N_{m}} \end{aligned}$$

### Comparative algorithms

In this study, to maintain conciseness, only brief summaries of each algorithm are presented in Table 3, instead of providing detailed explanations.

**Table 3:** A summary of the metaheuristic algorithms used in this paper.

Algorithm	Year	Summary
ECO	2024	Educational competition optimizer (ECO): A metaheuristic algorithm inspired by the competitive dynamics of educational systems, modeling student progression through elementary, middle, and high school stages^55^. These phases structure the shift from exploration to exploitation. ECO progressively refines the solution set over successive iterations, demonstrating robust performance on both benchmark tests and real-world applications.
HO	2024	Hippopotamus optimization algorithm (HO): A nature-inspired metaheuristic based on the behavioral patterns of hippopotamuses^56^. HO models candidate solutions through a trinary-phase system: positioning in water, defense, and predator evasion; to guide search dynamics. It achieves competitive results on 115 of 161 benchmark functions and excels in constrained engineering problems by effectively balancing exploration and exploitation.
OOA	2023	Osprey optimization algorithm (OOA): A bio-inspired metaheuristic based on the hunting strategy of ospreys^57^. OOA simulates prey detection, capture, and relocation phases to guide the search process through distinct exploration and exploitation stages. It demonstrates strong performance on CEC 2017 benchmark functions and outperforms 12 established algorithms in real-world constrained problems from the CEC 2011 suite.
ZOA	2022	Zebra optimization algorithm (ZOA): A bio-inspired metaheuristic based on the foraging and defensive behaviors of zebras^58^. ZOA guides the search process through simulated grazing and predator evasion, balancing exploration and exploitation. It outperforms nine well-known algorithms on 68 benchmark functions (including CEC2015/2017) and excels in real-world engineering design problems.

For consistency in benchmarking, all algorithms were configured with an identical population size of 50 and 1000 iterations. All simulations were conducted in MATLAB R2021a on a personal computer equipped with an Intel Core i5-3230M CPU at 2.60 GHz and 8.00 GB of RAM. Each algorithm was executed 30 times in independent runs. For the SFOA, the parameter *Gp* was set to 0.5, while for the ECO, the learning habit boundary was set to $H = 0.5$. The remaining algorithms were used in their original form without any control parameter adjustments, as they do not incorporate tunable control parameters. For the initial guess of secant method, $x_0$ is set to 0 and $x_1$ is set to $I_{ph}$.

## Results and discussion

### Convergence analysis

**Fig. 4:**
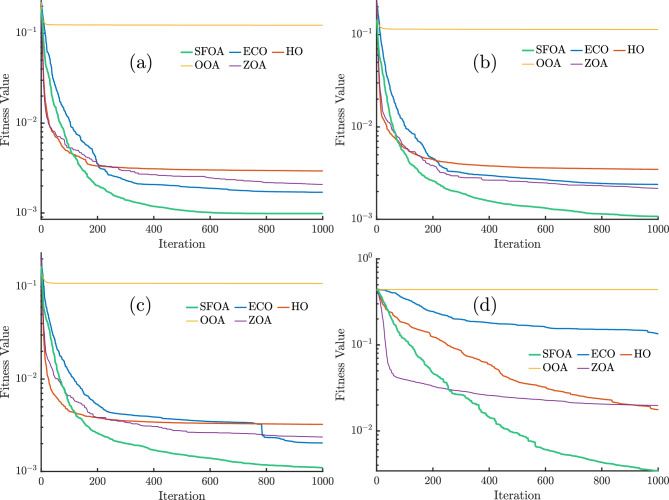
Convergence curves for (**a**) SDM, (**b**) DDM, (**c**) TDM, and (**d**) PVM for different algorithms without secant modification.

Figure 4 illustrates the convergence curves of the RMSE, without secant modification, for the SFOA, ECO, HO, OOA, and ZOA algorithms applied to the SDM, DDM, and TDM models, based on RTC France and PVM for the Photowatt-PWP201 module. Across all models, the SFOA algorithm demonstrates the fastest and most stable convergence, consistently reaching the lowest fitness values well before the maximum iteration limit, except in the case of PVM. For SDM, DDM, TDM, and PVM, SFOA achieves near-optimal solutions within the first 600–650 iterations, 800–850 iterations, 800–900 iterations, and 900–1000 iterations, respectively. Followed closely by ECO, ZOA, and HO, which converge more slowly and to slightly higher final fitness values.

OOA perform significantly worse across all cases, with OOA maintaining an almost constant and high fitness value throughout the iterations, indicating poor search capability and an inability to improve over time. The PVM case in Fig. 4d highlights these performance differences even more clearly; SFOA reaches the lowest fitness value with a sharp initial drop, while ECO, HO, and ZOA exhibit slower and less stable convergence patterns, and OOA once again shows negligible improvement.

**Fig. 5:**
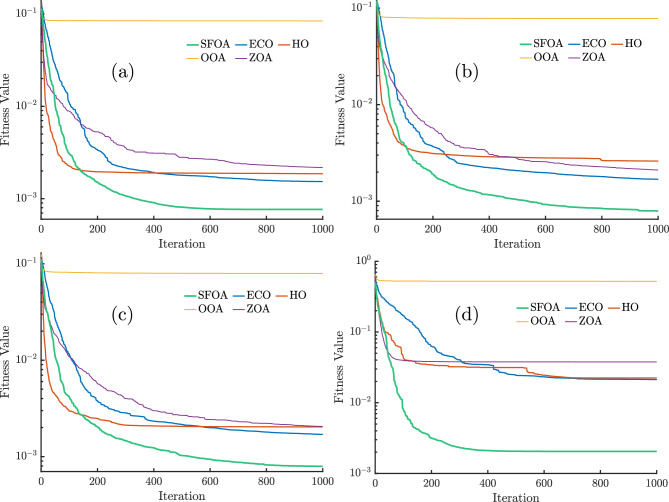
Convergence curves for (**a**) SDM, (**b**) DDM, (**c**) TDM, and (**d**) PVM for different algorithms with secant modification.

Figure 5 shows the convergence curves of the different algorithms for the various models in the case of modification with the secant method. The inclusion of the secant modification significantly accelerates convergence for all methods, with SFOA showing the most remarkable improvement. In this case, SFOA reaches the lowest fitness value, lower than $10^{-3}$, well before 750 iterations and maintains this advantage throughout the run. ECO and HO also benefit from the modification, converging faster and to lower final fitness values than in the non-secant case.

In contrast, ZOA experiences modest gains, converging more slowly and stabilizing at higher fitness levels, while OOA remains almost unchanged, stuck at a constant, poor fitness value. The PVM case in Fig. 5d highlights the advantage of the secant modification for SFOA, which quickly reaches near-optimal solutions, whereas other algorithms converge more gradually and to worse solutions.

**Fig. 6:**
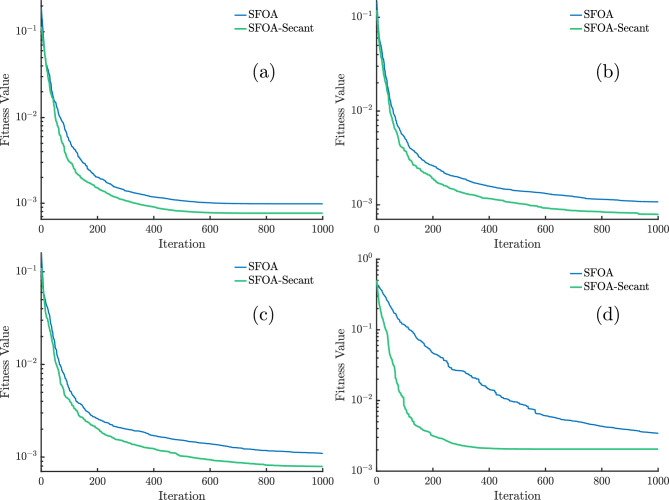
Convergence curves for SFOA with and without secant modification for (**a**) SDM, (**b**) DDM, (**c**) TDM, and (**d**) PVM.

To highlight the differences in terms of convergence and to provide a clearer view of the SFOA, Fig. 6 illustrates its convergence curves for the various models, in both cases with and without secant modification. Across all four models, the secant-enhanced SFOA (green curve) consistently converges faster and achieves lower fitness values than the standard version (blue curve).

### Accuracy of parameter extraction

Using five optimization algorithms and their corresponding secant-based hybrid variants, Table 4 summarizes the ideal parameters found for the SDM, DDM, and TDM based on the RTC France reference cell and the Photowatt-PWP201 PVM. The photocurrent $I_{ph}$, saturation current(s) ($I_{sd}$, $I_{sd1}$, $I_{sd2}$, $I_{sd3}$), shunt resistance $R_{sh}$, series resistance $R_{s}$, and ideality factor(s) (*n*, $n_1$, $n_2$, $n_3$), as well as the RMSE as a measure of fitting accuracy, are among the presented parameters for each model. The RMSE values of the secant-hybrid approaches are lower than those of their standalone counterparts, especially for the SDM and DDM. The most reliable accuracy across various PV models is provided by SFOA-Secant and ECO-Secant among the tested configurations, indicating the resilience of hybridization in parameter extraction.

The best results are obtained with the SFOA-Secant configuration for the TDM and DDM, yielding RMSE values of $7.3218\times 10^{-4}$ and $7.4162\times 10^{-4}$, respectively. These are closely followed by ECO-Secant for the DDM ($7.5727\times 10^{-4}$) and SFOA-Secant for the SDM ($7.6579\times 10^{-4}$). Such minimal error values indicate highly accurate parameter estimation and an excellent match between the modeled and experimental I–V characteristics. For the PVM, SFOA-Secant achieves the lowest RMSE of $2.0489\times 10^{-3}$.

In contrast, the poorest performance is observed for the OOA and OOA-Secant configurations in the PVM case, with RMSE values of $3.4675\times 10^{-1}$ and $1.9808\times 10^{-1}$, respectively, followed by OOA in the DDM ($2.4222\times 10^{-2}$) and OOA-Secant in the same model ($2.0863\times 10^{-2}$). These large deviations suggest that these settings fail to converge effectively to the optimal parameters, resulting in poor model fitting and significant mismatches with the measured data.

**Table 4:** Optimal parameters of three PV cell models (SDM, DDM, and TDM) based on RTC France reference cell, and PVM parameters for the Photowatt-PWP201.

	SFOA	ECO	HO	OOA	ZOA	SFOA-Secant	ECO-Secant	HO-Secant	OOA-Secant	ZOA-Secant
SDM										
$I_{ph}$(A)	0.76078	0.76073	0.76064	0.76207	0.76052	0.76079	0.76082	0.76049	0.76488	0.76061
$I_{sd}$ ($\mu$A)	0.32302	0.35435	0.27179	0.79519	0.30062	0.31039	0.29814	0.39037	0.22756	0.3168
$R_{sh}$ ($\Omega$)	53.7184	56.3764	51.9534	68.3279	55.3669	52.9648	51.8991	62.3637	57.206	56.4129
$R_{s}$ ($\Omega$)	0.036377	0.036006	0.037101	0.032865	0.036714	0.036553	0.036724	0.035612	0.041722	0.036585
*n*	1.4812	1.4906	1.4639	1.5779	1.4739	1.4772	1.4731	1.5005	1.4459	1.4792
RMSE	$9.8602\times 10^{-4}$	$1.0017\times 10^{-3}$	$1.0504\times 10^{-3}$	$2.3384\times 10^{-3}$	$1.0137\times 10^{-3}$	$7.6579\times 10^{-4}$	$7.6841\times 10^{-4}$	$8.6480\times 10^{-4}$	$4.9989\times 10^{-3}$	$7.8396\times 10^{-4}$
DDM										
$I_{ph}$(A)	0.760790	0.759770	0.761350	0.736970	0.761150	0.760830	0.760750	0.761190	0.791450	0.760860
$I_{sd1}$ ($\mu$A)	0.373630	0.071649	0.585210	0.854700	0.278570	0.187210	0.318150	0.126580	0.642170	0.208560
$I_{sd2}$ ($\mu$A)	0.271510	0.440680	0.169750	0.196520	0.196900	0.996220	0.267270	0.455080	0.965250	0.440740
$R_{sh}$ ($\Omega$)	54.479500	92.118600	45.177900	58.899000	49.559600	54.681700	54.089900	47.259800	12.189500	56.308800
$R_{s}$ ($\Omega$)	0.036555	0.035200	0.037444	0.036382	0.036542	0.037082	0.036714	0.037566	0.039587	0.036661
$n_1$	1.997700	1.859900	1.904200	1.707900	1.702700	1.434700	1.998900	1.407200	1.579900	1.448500
$n_2$	1.466400	1.514000	1.425800	1.486700	1.446000	2.000000	1.464500	1.746100	1.832100	1.799400
RMSE	$9.8346\times 10^{-4}$	$1.3697\times 10^{-3}$	$1.1097\times 10^{-3}$	$2.4222\times 10^{-2}$	$1.0235\times 10^{-3}$	$7.4192\times 10^{-4}$	$7.5727\times 10^{-4}$	$8.1849\times 10^{-4}$	$2.0863\times 10^{-2}$	$8.0037\times 10^{-4}$
TDM										
$I_{ph}$(A)	0.76077	0.76065	0.76031	0.76746	0.7607	0.76085	0.76058	0.76097	0.78249	0.76072
$I_{sd1}$ ($\mu$ A)	0.0043821	0.31689	0.73845	0.79243	0.35674	0.090904	0.93996	0.42128	0.43867	0.32592
$I_{sd2}$ ($\mu$A)	0.30437	0.1	0.10568	0.2784	0.18025	0.95898	0.24448	0.034704	0.43479	0.32714
$I_{sd3}$ ($\mu$A)	1	0.99067	0.27352	0.29601	0.44456	0.92695	0.98737	$3.38\times 10^{-5}$	0.32901	0.79995
$R_{sh}$ ($\Omega$)	54.4258	55.995	66.023	41.335	52.8921	55.8943	63.6227	55.9845	35.6222	64.1659
$R_{s}$ ($\Omega$)	0.036472	0.036265	0.0371	0.032955	0.037494	0.038114	0.0361	0.03503	0.039877	0.035866
$n_1$	2	1.4802	1.7654	1.7252	1.8137	1.3762	1.9902	1.5089	1.5551	1.4852
$n_2$	1.4757	1.9403	1.3978	1.5034	1.4318	1.9674	1.4603	1.9403	1.6355	1.9967
$n_3$	2.6576	4.6524	2.5093	3.1298	3.4027	2.008	4.6782	2.0347	2.7947	2.4205
RMSE	$9.8228\times 10^{-4}$	$9.8997\times 10^{-4}$	$1.0461\times 10^{-3}$	$4.3137\times 10^{-3}$	$1.0584\times 10^{-3}$	$7.3218\times 10^{-4}$	$8.3668\times 10^{-4}$	$9.5551\times 10^{-4}$	$1.5493\times 10^{-2}$	$9.2952\times 10^{-4}$
PVM										
$I_{ph}$(A)	1.0294	1.0241	1.0183	0.55027	1.0297	1.0314	1.0313	1.0284	0.90697	1.0215
$I_{sd}$ ($\mu$A)	4.0479	3.4115	2.4511	0.20772	6.1573	2.6276	2.7394	2.9996	0.00051304	1.3294
$R_{sh}$ ($\Omega$)	35.0831	975.7735	253.6792	550.5105	42.356	22.8261	23.5141	34.8845	342.7705	149.6894
$R_{s}$ ($\Omega$)	0.032981	0.034037	0.0349	0.025977	0.031502	0.034341	0.03421	0.034131	0.0005111	0.037522
*n*	1.3673	1.3479	1.3139	1.1652	1.4149	49.4484	49.6081	49.9476	30.3078	46.9245
RMSE	$2.4679\times 10^{-3}$	$3.9275\times 10^{-3}$	$4.9871\times 10^{-3}$	$3.4675\times 10^{-1}$	$2.9992\times 10^{-3}$	$2.0489\times 10^{-3}$	$2.0507\times 10^{-3}$	$2.1995\times 10^{-3}$	$1.9808\times 10^{-1}$	$4.6287\times 10^{-3}$

Table 5 shows that SFOA-Secant achieves the lowest RMSE across all PV models (SDM, DDM, TDM, and PVM), outperforming recent optimization methods reported in the literature. Several competing algorithms provide results only for simpler models, indicating limited scalability when handling higher-order PV models. Moreover, although some methods yield competitive RMSE values for SDM and DDM, their accuracy degrades for more complex models, particularly for PV modules. These results indicate that most existing approaches are constrained by the traditional RMSE-based objective formulation. In contrast, the proposed secant-based modification significantly enhances convergence accuracy and numerical stability, demonstrating that improving the problem formulation is essential for achieving consistently high-precision PV parameter extraction.

**Table 5:** Comparison of RMSE values obtained by the proposed SFOA-Secant method and state-of-the-art optimization algorithms for different PV models.

	SDM	DDM	TDM	PVM
SFOA-Secant	$7.6579 \times 10^{-4}$	$7.4192 \times 10^{-4}$	$7.3218 \times 10^{-4}$	$2.0489 \times 10^{-3}$
LaPSO^44^	$9.86022 \times 10^{-4}$	$9.82485 \times 10^{-4}$	–	$2.42507 \times 10^{-3}$
ASMA^45^	$9.8602 \times 10^{-4}$	$9.8248 \times 10^{-4}$	–	–
INFO^46^	$9.86022 \times 10^{-4}$	$9.82755 \times 10^{-4}$	$9.8297 \times 10^{-4}$	–
PSA^32^	$9.8602 \times 10^{-4}$	$9.7078 \times 10^{-4}$	–	–
NSGNDO^33^	$9.8602 \times 10^{-4}$	$9.8248 \times 10^{-4}$	–	$2.05296 \times 10^{-3}$
BDGOA^18^	$9.860218 \times 10^{-4}$	$9.82473 \times 10^{-4}$	$9.807313 \times 10^{-4}$	$2.425074 \times 10^{-3}$

### Performance metrics

Table 6 presents the performance metrics of the five optimization algorithms and their Secant-based hybrid variants in estimating the optimal parameters of the SDM, DDM, and TDM for RTC France reference cell, as well as the PVM for Photowatt-PWP201. The evaluation metrics include AE, MAE, MaxAE, MBE, and the average computation time $(avg \ t(s))$ as performance indicators. Across all models, the Secant-based variants generally outperform their standalone counterparts in terms of AE, MAE, and MaxAE, reflecting improved fitting precision. However, this accuracy gain often comes at the cost of increased computation time, particularly for the HO-Secant approach, which exhibits the longest runtimes in all cases.

The SFOA-Secant and ECO-Secant configurations consistently yield the best results. For instance, SFOA-Secant achieves some of the lowest values recorded for this model in the SDM case, with an AE of 0.017501793, an MAE of 0.000673146, and a MaxAE of 0.001528391. Similarly, SFOA-Secant produces an AE of 0.017008394 and an MAE of 0.000654169 for the DDM and an AE of 0.016697426 and an MAE of 0.000642209 for the TDM, both of which demonstrate extremely accurate parameter estimation. ECO-Secant outperforms all other methods in the PVM case, yielding the lowest AE of 0.04216968 and an MAE of 0.001686787.

The OOA, on the other hand, exhibits the worst results, especially in the PVM case, where AE reaches 7.61536214, MAE rises to 0.304614486, and MaxAE peaks at 0.481289424. Other models, like the DDM, also show this pattern, with OOA producing an AE of 0.602298626 and an MAE of 0.023165332. With AE values of 0.367535968 for the TDM and 4.133594452 for the PVM, OOA-Secant also performs poorly in some cases, showing that hybridization may not always lead to better performance when the base algorithm itself shows poor convergence characteristics.

**Table 6:** Performance of optimization algorithms and their secant variants for estimating optimal parameters of SDM, DDM, and TDM (RTC France cell) and PVM (Photowatt-PWP201) using AE, MAE, MaxAE, MBE, and average computation time.

	SFOA	ECO	HO	OOA	ZOA	SFOA-Secant	ECO-Secant	HO-Secant	OOA-Secant	ZOA-Secant
SDM										
AE	0.021526959	0.021622665	0.022845559	0.050763605	0.021943042	0.017501793	0.017638387	0.018732825	0.118481141	0.017357367
MAE	0.00082796	0.000831641	0.000878675	0.001952446	0.000843963	0.000673146	0.0006784	0.000720493	0.004556967	0.000667591
MaxAE	0.00250741	0.002511851	0.002483839	0.005511375	0.002495009	0.001528391	0.001499441	0.001803453	0.010509201	0.00173942
MBE	$-6.33\times 10^{-10}$	$-1.41\times 10^{-6}$	$2.85\times 10^{-6}$	$-9.01\times 10^{-5}$	$1.65\times 10^{-5}$	$1.67\times 10^{-8}$	$-1.08\times 10^{-6}$	$7.44\times 10^{-6}$	$-0.001413809$	$7.47\times 10^{-6}$
$avg \ t(s)$	1.088422943	1.970802227	10.04740036	2.31256186	2.331915777	6.807119133	15.7363276	46.62277743	22.51437215	17.71516555
DDM										
AE	0.021396248	0.029161283	0.023753331	0.602298626	0.022792662	0.017008394	0.017218986	0.018584321	0.468069529	0.017157213
MAE	0.000822933	0.001121588	0.00091359	0.023165332	0.000876641	0.000654169	0.000662269	0.000714782	0.018002674	0.000659893
MaxAE	0.002518657	0.002722071	0.002588921	0.034737274	0.002581361	0.001470803	0.001508008	0.001328667	0.041714532	0.001525407
MBE	$-1.10\times 10^{-6}$	$-3.29\times 10^{-6}$	$-3.99\times 10^{-7}$	0.017721513	$-1.26\times 10^{-6}$	$1.37\times 10^{-5}$	$4.21\times 10^{-6}$	$2.64\times 10^{-6}$	$-0.005188185$	$7.52\times 10^{-6}$
$avg \ t(s)$	1.138663217	1.537485893	10.28916127	2.500927227	2.452570877	9.95610725	16.60970476	54.65771759	26.50376938	19.76605558
TDM										
AE	0.021424183	0.021600032	0.022019404	0.095840015	0.022510246	0.016697426	0.01857127	0.020015556	0.367535968	0.01936447
MAE	0.000824007	0.00083077	0.0008469	0.003686154	0.000865779	0.000642209	0.00071428	0.000769829	0.014135999	0.000744787
MaxAE	0.002491293	0.002600323	0.00254561	0.007980916	0.002465977	0.001354346	0.001624849	0.002107019	0.023386899	0.002038388
MBE	$8.66\times 10^{-7}$	$3.38\times 10^{-5}$	$3.84\times 10^{-6}$	$-0.002204521$	$1.69\times 10^{-6}$	$-1.42\times 10^{-5}$	$-5.10\times 10^{-6}$	$4.86\times 10^{-6}$	$-0.008533525$	$6.33\times 10^{-6}$
$avg \ t(s)$	1.27819929	1.69401885	11.04641774	2.832161983	2.738773187	10.46314813	18.9287666	59.67902883	28.91716214	22.01212287
PVM										
AE	0.050044856	0.084451723	0.088742993	7.61536214	0.063282527	0.042492689	0.04216968	0.045636118	4.133594452	0.097091864
MAE	0.002001794	0.003378069	0.00354972	0.304614486	0.002531301	0.001699708	0.001686787	0.001825445	0.165343778	0.003883675
MaxAE	0.005067351	0.007404882	0.013384762	0.481289424	0.006221373	0.003822768	0.003925524	0.004282574	0.384427605	0.010249253
MBE	$-8.94\times 10^{-5}$	$-0.001519265$	0.00230622	0.250125902	$-1.20\times 10^{-5}$	$-1.07\times 10^{-7}$	$9.76\times 10^{-8}$	$3.81\times 10^{-6}$	$-0.030787725$	$-4.50\times 10^{-5}$
$avg \ t(s)$	1.308052823	1.74788223	11.8237347	2.899556927	2.823254947	11.61623352	12.9419986	46.16202316	32.60266903	21.57842688

From the average computation time reported in Table 6, it is evident that all Secant-based variants require a longer execution time compared to their conventional counterparts. This increase is attributed to the additional iterative root-finding process introduced by the Secant method during objective-function evaluation. The effect is consistent across all PV models and optimization algorithms. These results experimentally confirm the computational time approximation formulated in (28), demonstrating that the improved accuracy achieved by the Secant-based formulation is obtained at the cost of increased computational effort.

### Robustness and repeatability

The statistical analysis of RMSE values for the SDM, DDM, TDM, and PVM based on 30 independent runs using five optimization algorithms and their Secant-hybrid variants is shown in Table 7. Insight into the accuracy and consistency of the methods is provided by reporting the minimum, maximum, mean, median, and standard deviation for each case.

The findings show that the Secant-hybrid versions, SFOA-Secant in particular, achieve consistently low RMSE values with little variation across all PV models. For example, SFOA-Secant exhibits near-perfect repeatability in the SDM, maintaining an exceptionally stable RMSE of roughly $7.6579\times 10^{-4}$ with a standard deviation of just $1.3793\times 10^{-9}$. Both SFOA-Secant and ECO-Secant demonstrate their robustness in parameter extraction by achieving low mean RMSE values and small standard deviations in the DDM and TDM.

In contrast, specific baseline algorithms exhibit substantial variability and higher error values. Notably, OOA shows fluctuations, as observed in the SDM (mean RMSE of $1.2286\times 10^{-1}$, maximum of $2.5212\times 10^{-1}$) and PVM (mean RMSE of $4.4056\times 10^{-1}$). In the PVM case, the standard deviation for OOA reaches $1.7721\times 10^{-2}$, indicating a strong sensitivity to initialization or being stuck in local minima. By comparison, the best-performing hybrid variants consistently combine accuracy with stability, making them more suitable for reliable PV model parameter estimation.

**Table 7:** Statistical comparison of RMSE values for SDM, DDM, TDM, and PVM obtained with different algorithms after 30 runs.

	SFOA	ECO	HO	OOA	ZOA	SFOA-Secant	ECO-Secant	HO-Secant	OOA-Secant	ZOA-Secant
SDM										
Min	$9.8602\times 10^{-4}$	$1.0017\times 10^{-3}$	$1.0504\times 10^{-3}$	$2.3384\times 10^{-3}$	$1.0137\times 10^{-3}$	$7.6579\times 10^{-4}$	$7.6841\times 10^{-4}$	$8.6480\times 10^{-4}$	$4.9989\times 10^{-3}$	$7.8396\times 10^{-4}$
Max	$9.8642\times 10^{-4}$	$2.4480\times 10^{-3}$	$1.1089\times 10^{-2}$	$2.5212\times 10^{-1}$	$4.8537\times 10^{-3}$	$7.6579\times 10^{-4}$	$4.7728\times 10^{-3}$	$4.1313\times 10^{-3}$	$1.3457\times 10^{-1}$	$4.9543\times 10^{-3}$
Mean	$9.8604\times 10^{-4}$	$1.6998\times 10^{-3}$	$2.9335\times 10^{-3}$	$1.2286\times 10^{-1}$	$2.0763\times 10^{-3}$	$7.6579\times 10^{-4}$	$1.5360\times 10^{-3}$	$1.8642\times 10^{-3}$	$8.4089\times 10^{-2}$	$2.1789\times 10^{-3}$
Median	$9.8602\times 10^{-4}$	$1.7633\times 10^{-3}$	$2.1204\times 10^{-3}$	$1.2766\times 10^{-1}$	$1.5894\times 10^{-3}$	$7.6579\times 10^{-4}$	$1.4360\times 10^{-3}$	$1.7141\times 10^{-3}$	$9.0294\times 10^{-2}$	$1.8889\times 10^{-3}$
Std	$7.2168\times 10^{-8}$	$4.6461\times 10^{-4}$	$2.3200\times 10^{-3}$	$6.7369\times 10^{-2}$	$1.2019\times 10^{-3}$	$1.3793\times 10^{-9}$	$6.9959\times 10^{-4}$	$7.4266\times 10^{-4}$	$3.3748\times 10^{-2}$	$1.0688\times 10^{-3}$
DDM										
Min	$9.8346\times 10^{-4}$	$1.3697\times 10^{-3}$	$1.1097\times 10^{-3}$	$2.4222\times 10^{-2}$	$1.0235\times 10^{-3}$	$7.4192\times 10^{-4}$	$7.5727\times 10^{-4}$	$8.1849\times 10^{-4}$	$2.0863\times 10^{-2}$	$8.0037\times 10^{-4}$
Max	$2.1563\times 10^{-3}$	$5.5725\times 10^{-3}$	$9.2998\times 10^{-3}$	$2.8580\times 10^{-1}$	$3.8739\times 10^{-3}$	$1.1829\times 10^{-3}$	$2.9951\times 10^{-3}$	$6.9363\times 10^{-3}$	$1.3027\times 10^{-1}$	$4.7242\times 10^{-3}$
Mean	$1.0741\times 10^{-3}$	$2.3881\times 10^{-3}$	$3.4729\times 10^{-3}$	$1.1366\times 10^{-1}$	$2.1656\times 10^{-3}$	$7.9045\times 10^{-4}$	$1.6881\times 10^{-3}$	$2.6015\times 10^{-3}$	$7.8022\times 10^{-2}$	$2.1048\times 10^{-3}$
Median	$9.9153\times 10^{-4}$	$2.3455\times 10^{-3}$	$2.7147\times 10^{-3}$	$1.1148\times 10^{-1}$	$2.2971\times 10^{-3}$	$7.6588\times 10^{-4}$	$1.6404\times 10^{-3}$	$2.3096\times 10^{-3}$	$8.0293\times 10^{-2}$	$1.9065\times 10^{-3}$
Std	$2.2741\times 10^{-4}$	$7.4026\times 10^{-4}$	$2.3939\times 10^{-3}$	$5.8097\times 10^{-2}$	$7.1293\times 10^{-4}$	$8.2645\times 10^{-5}$	$5.3134\times 10^{-4}$	$1.4994\times 10^{-3}$	$2.9673\times 10^{-2}$	$9.8916\times 10^{-4}$
TDM										
Min	$9.8228\times 10^{-4}$	$9.8997\times 10^{-4}$	$1.0461\times 10^{-3}$	$4.3137\times 10^{-3}$	$1.0584\times 10^{-3}$	$7.3218\times 10^{-4}$	$8.3668\times 10^{-4}$	$9.5551\times 10^{-4}$	$1.5493\times 10^{-2}$	$9.2952\times 10^{-4}$
Max	$1.6592\times 10^{-3}$	$4.0512\times 10^{-3}$	$9.3912\times 10^{-3}$	$2.5878\times 10^{-1}$	$4.3768\times 10^{-3}$	$1.4869\times 10^{-3}$	$2.2642\times 10^{-3}$	$4.1855\times 10^{-3}$	$1.4083\times 10^{-1}$	$6.0019\times 10^{-3}$
Mean	$1.0972\times 10^{-3}$	$2.0353\times 10^{-3}$	$3.2334\times 10^{-3}$	$1.0832\times 10^{-1}$	$2.3598\times 10^{-3}$	$7.9207\times 10^{-4}$	$1.6998\times 10^{-3}$	$2.0317\times 10^{-3}$	$7.9420\times 10^{-2}$	$2.0518\times 10^{-3}$
Median	$1.0067\times 10^{-3}$	$1.9752\times 10^{-3}$	$2.5084\times 10^{-3}$	$1.0122\times 10^{-1}$	$2.2016\times 10^{-3}$	$7.6062\times 10^{-4}$	$1.7726\times 10^{-3}$	$1.8024\times 10^{-3}$	$7.4115\times 10^{-2}$	$1.9108\times 10^{-3}$
Std	$1.7174\times 10^{-4}$	$6.5930\times 10^{-4}$	$1.9629\times 10^{-3}$	$6.4831\times 10^{-2}$	$8.8253\times 10^{-4}$	$1.3551\times 10^{-4}$	$3.8192\times 10^{-4}$	$8.2476\times 10^{-4}$	$3.0786\times 10^{-2}$	$9.3487\times 10^{-4}$
PVM										
Min	$2.4679\times 10^{-3}$	$3.9275\times 10^{-3}$	$4.9871\times 10^{-3}$	$3.4675\times 10^{-1}$	$2.9992\times 10^{-3}$	$2.0489\times 10^{-3}$	$2.0507\times 10^{-3}$	$2.1995\times 10^{-3}$	$1.9808\times 10^{-1}$	$4.6287\times 10^{-3}$
Max	$6.6471\times 10^{-3}$	$2.7437\times 10^{-1}$	$5.7407\times 10^{-2}$	$4.4414\times 10^{-1}$	$8.0658\times 10^{-2}$	$2.0489\times 10^{-3}$	$2.7425\times 10^{-1}$	$2.7425\times 10^{-1}$	$7.2904\times 10^{-1}$	$2.7425\times 10^{-1}$
Mean	$3.4375\times 10^{-3}$	$1.3438\times 10^{-1}$	$1.7650\times 10^{-2}$	$4.4056\times 10^{-1}$	$1.9795\times 10^{-2}$	$2.0489\times 10^{-3}$	$2.1254\times 10^{-2}$	$2.2362\times 10^{-2}$	$5.2181\times 10^{-1}$	$3.7700\times 10^{-2}$
Median	$3.1716\times 10^{-3}$	$8.4014\times 10^{-2}$	$1.4038\times 10^{-2}$	$4.4387\times 10^{-1}$	$4.7669\times 10^{-3}$	$2.0489\times 10^{-3}$	$2.5336\times 10^{-3}$	$3.5901\times 10^{-3}$	$5.3402\times 10^{-1}$	$1.1744\times 10^{-2}$
Std	$8.7257\times 10^{-4}$	$1.2757\times 10^{-1}$	$1.2385\times 10^{-2}$	$1.7721\times 10^{-2}$	$2.7998\times 10^{-2}$	$4.9140\times 10^{-10}$	$6.8808\times 10^{-2}$	$6.8499\times 10^{-2}$	$1.2964\times 10^{-1}$	$8.0317\times 10^{-2}$

**Fig. 7:**
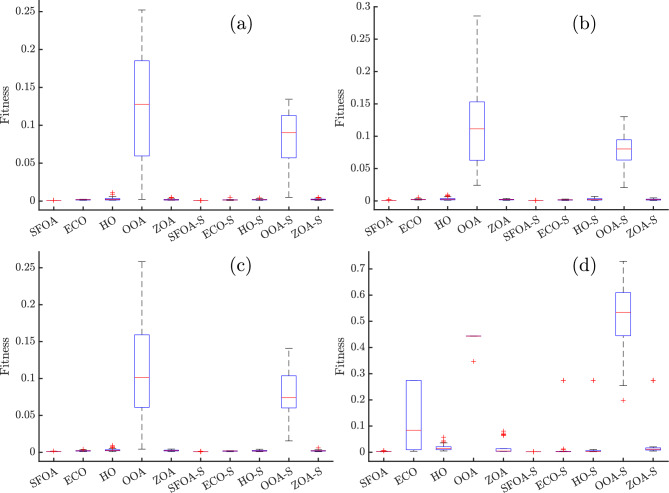
Box plot for (**a**) SDM, (**b**) DDM, (**c**) TDM, and (**d**) PVM for different algorithms with and without secant modification.

Figure 7 presents box plots of the fitness for the different optimization algorithms, with and without the secant modification, applied to SDM, DDM, TDM, and PVM. Across all subfigures, the Secant-based variants, especially SFOA-Secant exhibit extremely compact distributions with minimal interquartile range (IQR) and negligible outliers, indicating both high accuracy and exceptional stability. In particular, SFOA-Secant maintains median RMSE values close to zero with no visible spread, confirming its repeatable performance across independent runs.

In contrast, specific baseline algorithms, such as OOA and HO, show significant variance and higher median RMSE values, as clearly visible in the elongated box and whiskers, along with numerous outliers. This effect is most pronounced in SDM of Fig. 7a and TDM in Fig. 7c, where OOA’s performance fluctuates considerably between runs. For PVM in Fig. 7d, OOA and ECO display substantial dispersion, whereas SFOA-Secant, ECO-Secant, and other hybrid variants retain consistently low error values.

Therefore, the figure graphically supports the previous statistical conclusions: the Secant-hybrid methods, particularly SFOA-Secant, are consistently the most accurate and dependable, yielding strongly clustered, low-error results for all PV models.

### Statistical validation

The p-values from the Wilcoxon rank-sum test, which compares the RMSE distributions of SFOA-Secant to each of the other tested algorithms for SDM, DDM, TDM, and PVM, are shown in Table 8. At the $5\%$ significance level, SFOA-Secant significantly outperforms the compared method, as indicated by the p-values, which are incredibly small in all cases and range from $10^{-10}$ to $10^{-11}$, accompanied by a “+” symbol. The robustness and superiority of the SFOA-Secant approach in obtaining lower RMSE values with high confidence are highlighted by this consistent statistical dominance across all models and algorithms.

**Table 8:** Wilcoxon rank-sum test *p*-values comparing SFOA-Secant with other optimization algorithms for SDM, DDM, TDM, and PVM models.

SFOA-Secant vs.	SDM		DDM		TDM		PVM	
SFOA	$3.0199 \times 10^{-11}$	+	$3.82 \times 10^{-10}$	+	$5.07 \times 10^{-10}$	+	$3.02 \times 10^{-11}$	+
ECO	$3.0199 \times 10^{-11}$	+	$3.02 \times 10^{-11}$	+	$5.49 \times 10^{-11}$	+	$3.02 \times 10^{-11}$	+
HO	$3.0199 \times 10^{-11}$	+	$3.69 \times 10^{-11}$	+	$4.50 \times 10^{-11}$	+	$3.02 \times 10^{-11}$	+
OOA	$3.0199 \times 10^{-11}$	+	$3.02 \times 10^{-11}$	+	$3.02 \times 10^{-11}$	+	$3.02 \times 10^{-11}$	+
ZOA	$3.0199 \times 10^{-11}$	+	$3.69 \times 10^{-11}$	+	$4.98 \times 10^{-11}$	+	$3.02 \times 10^{-11}$	+
ECO-Secant	$3.0199 \times 10^{-11}$	+	$3.47 \times 10^{-10}$	+	$9.92 \times 10^{-11}$	+	$3.02 \times 10^{-11}$	+
HO-Secant	$3.0199 \times 10^{-11}$	+	$6.70 \times 10^{-11}$	+	$8.15 \times 10^{-11}$	+	$3.02 \times 10^{-11}$	+
OOA-Secant	$3.0199 \times 10^{-11}$	+	$3.02 \times 10^{-11}$	+	$3.02 \times 10^{-11}$	+	$3.02 \times 10^{-11}$	+
ZOA-Secant	$3.0199 \times 10^{-11}$	+	$1.09 \times 10^{-10}$	+	$5.49 \times 10^{-11}$	+	$3.02 \times 10^{-11}$	+

Table 9 presents the results of the Friedman ranking test, showing the mean ranks and the corresponding rank orders for each algorithm across all four PV models. In every case, SFOA-Secant obtains the best possible mean rank (close to 1.0) and is ranked first, confirming its consistent top performance. The second-best performers vary depending on the model, SFOA in SDM, DDM, and TDM, and ECO-Secant in PVM; although their mean ranks are notably higher than SFOA-Secant’s. Conversely, OOA and OOA-Secant occupy the bottom ranks in nearly all cases, reflecting their poor optimization stability and accuracy observed in earlier tables.

**Table 9:** Mean and order rankings from the Friedman test of different tested algorithms for SDM, DDM, TDM, and PVM.

	SFOA	ECO	HO	OOA	ZOA	SFOA-Secant	ECO-Secant	HO-Secant	OOA-Secant	ZOA-Secant
SDM										
Mean rank	2.1666	5.3333	6.4666	9.6666	5.3334	1	4.4333	5.5	9.2666	5.8333
Rank order	2	4	8	10	5	1	3	6	9	7
DDM										
Mean rank	2.3333	5.9333	6.1666	9.6666	5.2666	1.0666	4.4666	5.5666	9.3333	5.2
Rank order	2	7	8	10	5	1	3	6	9	4
TDM										
Mean rank	2.4	5.3333	6.6	9.6	5.7	1.1	4.4333	5.2333	9.4	5.2
Rank order	2	6	8	10	7	1	3	5	9	4
PVM										
Mean rank	3.0666	7	6.5666	9.2333	4.9666	1	3.0333	4.1333	9.7666	6.2333
Rank order	3	8	7	9	5	1	2	4	10	6

With results that are statistically significant when compared to all competitors, SFOA-Secant is not only the most accurate configuration but also the most consistently dominant, according to the statistical tests, which generally support the conclusions from the error analysis.

### Characteristic curves

**Fig. 8:**
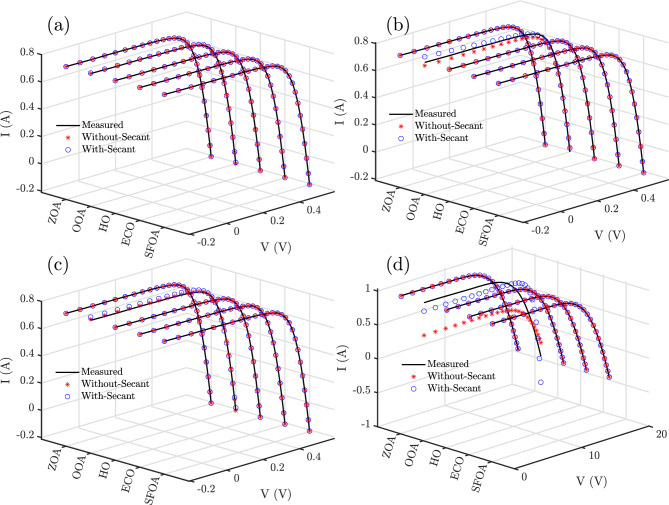
The I–V characteristic curves of RTC France based on (**a**) SDM, (**b**) DDM, and (**c**) TDM and Photowatt-PWP201 based on (**d**) PVM using different algorithms.

**Fig. 9:**
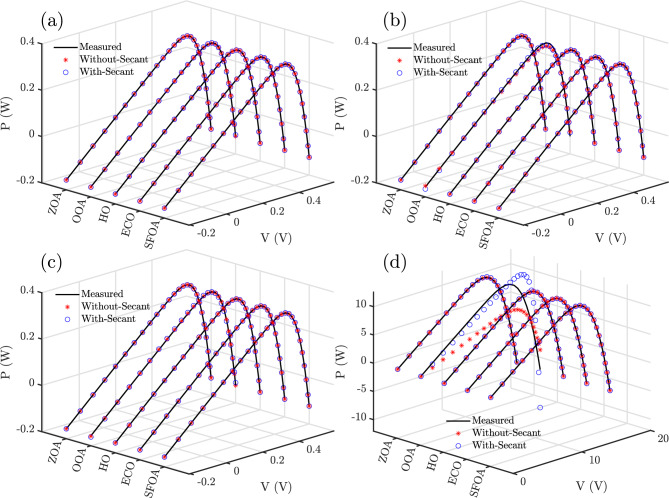
The P–V characteristic curves of RTC France based on (**a**) SDM, (**b**) DDM, and (**c**) TDM and Photowatt-PWP201 based on (**d**) PVM using different algorithms.

Figures 8 and 9 present the I–V and P–V characteristic curves, respectively, for RTC France based on SDM, DDM, TDM, and Photowatt-PWP201 based on PVM, using different algorithms with and without the secant modification. In both sets of plots, the measured experimental curves serve as the reference, while the estimated curves from each optimization approach are plotted for comparison.

Across the first three models, Figs. 8a–c and 9a–c, the secant-based variants, especially SFOA-Secant achieve almost perfect overlap with the measured curves, demonstrating precise parameter estimation and accurate reproduction of both the current and power profiles. The differences between the measured and estimated curves are practically imperceptible for these cases, indicating negligible modeling error.

In contrast, some baseline algorithms, particularly OOA for PVM, exhibit noticeable deviations. The secant modification effectively reduces these discrepancies, with SFOA-Secant and ECO-Secant producing near-perfect fits even in the more challenging PVM case.

These figures support the previous statistical and error-metric findings; the Secant-based hybrids, particularly SFOA-Secant, exhibit the lowest numerical errors and the highest fidelity in accurately representing the actual electrical behavior of the PV models under all operating conditions.

## Conclusion

The extraction of unknown parameters in photovoltaic (PV) models is a fundamental task, as it enables a rigorous, physics-based interpretation of PV cell and module behavior. Such an understanding directly supports practical applications, including accurate performance prediction, real-time monitoring, fault diagnosis, and the development of effective strategies for system optimization and efficiency enhancement. In this paper, the parameters of several PV models were extracted using a recently developed metaheuristic, namely the Starfish Optimization Algorithm (SFOA). Its performance was systematically evaluated and compared with four recent state-of-the-art algorithms: the Educational Competition Optimizer (ECO), Hippopotamus Optimization Algorithm (HO), Osprey Optimization Algorithm (OOA), and Zebra Optimization Algorithm (ZOA). Two objective function formulations were investigated: the conventional RMSE-based formulation and a novel secant-based reformulation. The primary contribution of this work lies in the development and successful integration of the secant-based objective function into the PV parameter extraction problem.

The results demonstrate that the secant-hybrid variants consistently achieve lower RMSE values with negligible variance across all considered PV models. An ablation analysis, conducted by replacing the proposed secant-based objective with the classical RMSE formulation while keeping all optimization settings unchanged, confirms that the observed performance gains originate from the proposed reformulation rather than from algorithmic parameter tuning. Furthermore, statistical validation using the Wilcoxon rank-sum test verifies that the improvements are statistically significant and not attributable to random effects.

Among all tested configurations, the SFOA-Secant approach exhibits the fastest and most stable convergence behavior, reliably attaining the lowest fitness values well before reaching the maximum number of iterations. The best overall results were obtained using SFOA-Secant for the three-diode model (TDM) and double-diode model (DDM), achieving RMSE values of $7.3218 \times 10^{-4}$ and $7.4162 \times 10^{-4}$, respectively. These results are closely followed by ECO-Secant for the DDM ($7.5727 \times 10^{-4}$) and SFOA-Secant for the single-diode model (SDM) ($7.6579 \times 10^{-4}$). Comprehensive statistical analyses further confirm that SFOA-Secant consistently outperforms all competing methods with high precision and robustness across different PV models.

In contrast, the OOA and OOA-Secant configurations exhibit inferior performance in most scenarios, particularly for the PV module (PVM) case. Although the secant-based hybridization improves accuracy in some instances, it also introduces additional computational overhead. This increase in computation time is mainly attributed to the iterative root-finding process embedded within the objective function evaluation. Moreover, the I–V and P–V characteristic curves obtained using OOA, with and without secant modification, show noticeable deviations from the experimental data, indicating poor convergence behavior.

The main limitation of the proposed secant-based objective function lies in its increased computational complexity, as evidenced by the higher average runtimes observed across different algorithms. While this overhead is acceptable for offline parameter identification, it may restrict the direct applicability of the method in strict real-time or embedded environments without further optimization.

Future research can address these limitations through several promising directions.

- The proposed secant-based objective function can be evaluated on additional benchmark datasets and under varying environmental conditions to further assess its generalization capability.
- Computational efficiency may be improved by developing adaptive or simplified variants of the secant method that preserve accuracy while reducing runtime.
- Enhancing poorly performing algorithms, such as OOA, particularly for PV module parameter extraction, represents another important research avenue.
- The real-time implementation of SFOA-based parameter extraction in operational PV systems, possibly through hardware acceleration or reduced-order modeling, constitutes a valuable direction for future work.

## Data Availability

All data generated or analyzed during this study are included in this published article.

## References

[1] Rumbayan, M., Kindangen, J., Sambul, A., Sompie, S. & Cross, J. Solar energy implementation in rural communities and its contributions to SDGS: A systematic literature review. *Unconvent. Resour.* **6**, 100180. 10.1016/j.uncres.2025.100180 (2025).

[2] Osei, J., Brown, K. & Nejati, M. Understanding the factors affecting social acceptance of solar energy technologies. *Energy Strat. Rev.* **61**, 101861. 10.1016/j.esr.2025.101861 (2025).

[3] Imasiku, K. Comprehensive approaches to electrifying rural health facilities: Integrating renewable energy and financial mechanisms in sub-saharan africa. *Energy Strat. Revi.* **59**, 101736. 10.1016/j.esr.2025.101736 (2025).

[4] Li, S., Fang, Z., Verma, S. C., Wei, J. & Savkin, A. V. Navigation and deployment of solar-powered unmanned aerial vehicles for civilian applications: A comprehensive review. *Drones* **8**, 42. 10.3390/drones8020042 (2024).

[5] Wu, J., Zhang, Y., Zhu, L. & Li, H. Residential solar photovoltaic adoption: An in-depth review on potential, main barriers and related incentives. *Energy Build.* **339**, 115766. 10.1016/j.enbuild.2025.115766 (2025).

[6] Balci, Y. & Erbay, C. Harnessing solar energy for sustainable green hydrogen production in türkiye: Opportunities, and economic viability. *Int. J. Hydrog. Energy* **87**, 985–996. 10.1016/j.ijhydene.2024.09.098 (2024).

[7] Mathew, T. C. & Nagaraja Pandian, S. Unveiling the shadows: A qualitative exploration of barriers to rooftop solar photovoltaic adoption in residential sectors. *Clean Energy* **8**, 218–228. 10.1093/ce/zkae065 (2024).

[8] Bouali, Y. & Alamri, B. Enhancing radial distribution system performance through optimal allocation and sizing of photovoltaic and wind turbine distribution generation units with rüppell’s fox optimizer. *Mathematics* **13**, 2399. 10.3390/math13152399 (2025).

[9] Ali, A. O. et al. Advancements in photovoltaic technology: A comprehensive review of recent advances and future prospects. *Energy Conv. Manag.: X* **26**, 100952. 10.1016/j.ecmx.2025.100952 (2025).

[10] Talukder, M. J. Advances in high-efficiency solar photovoltaic materials: A comprehensive review of perovskite and tandem cell technologies. *SSRN Electr. J.*10.2139/ssrn.5192349 (2025).

[11] Mdallal, A. et al. A comprehensive review on solar photovoltaics: Navigating generational shifts, innovations, and sustainability. *Sustain. Horiz.* **13**, 100137. 10.1016/j.horiz.2025.100137 (2025).

[12] Kumar Singla, M., Gupta, J., Safaraliev, M., Zeinoddini-Meymand, H. & Ghanizadeh, A. J. Mathematical modeling for solar cell optimization. Evaluating sustainability with different diode configurations*IEEE Access* **12**, 93802–93822. 10.1109/access.2024.3424416 (2024).

[13] Bouali, Y. & Alamri, B. Parameter extraction for photovoltaic models with flood-algorithm-based optimization. *Mathematics* **13**, 19. 10.3390/math13010019 (2024).

[14] Gu, Z., Xiong, G. & Fu, X. Parameter extraction of solar photovoltaic cell and module models with metaheuristic algorithms: A review. *Sustainability* **15**, 3312. 10.3390/su15043312 (2023).

[15] El Marghichi, M. et al. Accurate extraction of electrical parameters in three-diode photovoltaic systems through the enhanced mother tree methodology: A novel approach for parameter estimation. *PLoS ONE* **20**, e0318575. 10.1371/journal.pone.0318575 (2025). 40036287 10.1371/journal.pone.0318575 PMC11878931

[16] Khalifa, H. et al. Parameter extraction of pv models under varying meteorological conditions using a modified electric eel foraging optimization algorithm. *Sci. Rep.* 10.1038/s41598-025-98270-y (2025) PMC12130207 40456883

[17] Wang, W. & Tian, J. Optimal parameters extraction for photovoltaic models utilizing an artificial rabbit optimizer incorporating swarm-elite learning mechanism’s levy flight strategy. *Comput. Electr. Eng.* **127**, 110582. 10.1016/j.compeleceng.2025.110582 (2025).

[18] Jabari, M. et al. Parameter identification of pv solar cells and modules using bio dynamics grasshopper optimization algorithm. *IET Gener. Trans. Distrib.* **18**: 3314–3338, 10.1049/gtd2.13279 (2024).

[19] Izci, D., Ekinci, S. & Hussien, A. G. Efficient parameter extraction of photovoltaic models with a novel enhanced prairie dog optimization algorithm. *Sci. Rep.* **14**, 10.1038/s41598-024-58503-y (2024). 10.1038/s41598-024-58503-y PMC10995185 38575704

[20] Ali, H. H. et al. A new hybrid multi-population GTO-BWO approach for parameter estimation of photovoltaic cells and modules. *Sustainability* **15**, 11089. 10.3390/su151411089 (2023).

[21] Mohamed, R. et al. Novel hybrid Kepler optimization algorithm for parameter estimation of photovoltaic modules. *Sci. Rep.*10.1038/s41598-024-52416-6 (2024). 38342929 10.1038/s41598-024-52416-6 PMC10859389

[22] Sharma, P., Raju, S., Salgotra, R. & Gandomi, A. H. Parametric estimation of photovoltaic systems using a new multi-hybrid evolutionary algorithm. *Energy Rep.* **10**, 4447–4464. 10.1016/j.egyr.2023.11.012 (2023).

[23] Almutairi, S. Z. & Shaheen, A. M. A novel kangaroo escape optimizer for parameter estimation of solar photovoltaic cells/modules via one, two and three-diode equivalent circuit modeling. *Sci. Rep.*10.1038/s41598-025-19917-4 (2025). 40987918 10.1038/s41598-025-19917-4 PMC12457617

[24] Çelik, E. et al. Reconfigured single- and double-diode models for improved modelling of solar cells/modules. *Sci. Rep.*10.1038/s41598-025-86063-2 (2025). 10.1038/s41598-025-86063-2 PMC11735614 39814860

[25] Parida, S. M. et al. Optimal parameter identification of photovoltaic systems based on enhanced differential evolution optimization technique. *Sci. Rep.* **15**, 10.1038/s41598-025-85115-x (2025). 10.1038/s41598-025-85115-x PMC11739471 39820510

[26] Saleh Alluhaidan, A. et al. Refined photovoltaic parameters estimation via an improved sinh cosh optimizer with trigonometric operators. *Sci. Rep.* **15**, 10.1038/s41598-025-85841-2 (2025). 10.1038/s41598-025-85841-2 PMC11802759 39915523

[27] Abazine, I. et al. Accurate parameter extraction for photovoltaic systems using an improved and stable hybrid analytical-NRBO algorithm. *Int. J. Hydrog.Energy* **139**, 564–593. 10.1016/j.ijhydene.2025.05.208 (2025).

[28] Kumar, A., Sharma, T., Singh, M. K., Pant, S. & Sharma, H. K. Photovoltaic module parameter estimation by using wild horse optimizer. *Procedia Comput. Sci.* **258**, 2808–2816. 10.1016/j.procs.2025.04.541 (2025).

[29] Liu, E.-J., Chen, R.-W., Wang, Q.-A. & Lu, W.-L. Shuffled puma optimizer for parameter extraction and sensitivity analysis in photovoltaic models. *Energies* **18**, 4008. 10.3390/en18154008 (2025).

[30] Dal, S. & Sezgin, N. Estimation of uncertain parameters in single and double diode models of photovoltaic panels using frilled lizard optimization. *Electronics* **14**, 796. 10.3390/electronics14040796 (2025).

[31] Li, Q., Zhou, Y. & Luo, Q. Scso: snake optimization with sine-cosine algorithm for parameter extraction of solar photovoltaic models. *Discov. Appl. Sci.* **7**, 10.1007/s42452-025-06756-1 (2025).

[32] Bennagi, A., AlHousrya, O., Cotfas, D. T. & Cotfas, P. A. Parameter extraction of photovoltaic cells and panels using a pid-based metaheuristic algorithm. *Appl. Sci.* **15**, 7403. 10.3390/app15137403 (2025).

[33] Ghetas, M. & Elshourbagy, M. Parameters extraction of photovoltaic models using enhanced generalized normal distribution optimization with neighborhood search. *Neural Comput. Appl.* **36**, 14035–14052. 10.1007/s00521-024-09609-x (2024).

[34] Ajay Rathod, A. & Subramanian, B. Efficient approach for optimal parameter estimation of pv using pelican optimization algorithm. *Cogent Eng.* **11**, 10.1080/23311916.2024.2380805 (2024).

[35] Kumari, P. A. et al. Application of dso algorithm for estimating the parameters of triple diode model-based solar pv system. *Sci. Rep.* **14**, 10.1038/s41598-024-53582-3 (2024). 10.1038/s41598-024-53582-3 PMC10873396 38365987

[36] Khajuria, R. et al. *An optimized solar PV model parameter extraction technique using lungs performance-based optimization*, 399–410 (Springer Nature, Singapore, 2025).

[37] Khajuria, R., Sharma, P., Lamba, R., Kumar, R. & Raju, S. *Model Parameter Extraction of Solar PV Cell Using Gold Rush Optimizer*, 163–173 (Springer Nature Singapore, 2024).

[38] Hussain, M. T. et al. Archimedes optimization algorithm based parameter extraction of photovoltaic models on a decent basis for novel accurate rmse calculation. *Front. Energy Res.* **11**, 10.3389/fenrg.2023.1326313 (2024).

[39] Chen, X., Wang, S. & He, K. Parameter estimation of various pv cells and modules using an improved simultaneous heat transfer search algorithm. *J. Comput. Electron.* **23**, 584–599. 10.1007/s10825-024-02153-w (2024).

[40] Ru, X. Parameter extraction of photovoltaic model based on butterfly optimization algorithm with chaos learning strategy. *Solar Energy* **269**, 112353. 10.1016/j.solener.2024.112353 (2024).

[41] El-Dabah, M. A., El-Sehiemy, R. A., Hasanien, H. M. & Saad, B. Photovoltaic model parameters identification using northern goshawk optimization algorithm. *Energy* **262**, 125522. 10.1016/j.energy.2022.125522 (2023).

[42] Beşkirli, A. & Dağ, İ. Parameter extraction for photovoltaic models with tree seed algorithm. *Energy Rep.* **9**, 174–185. 10.1016/j.egyr.2022.10.386 (2023).

[43] Duan, Z., Yu, H., Zhang, Q. & Tian, L. Parameter extraction of solar photovoltaic model based on nutcracker optimization algorithm. *Appl. Sci.* **13**, 6710. 10.3390/app13116710 (2023).

[44] Li, Y., Yu, K., Liang, J., Yue, C. & Qiao, K. A landscape-aware particle swarm optimization for parameter identification of photovoltaic models. *Appl. Soft Comput.* **131**, 109793. 10.1016/j.asoc.2022.109793 (2022).

[45] Lin, H. et al. Adaptive slime mould algorithm for optimal design of photovoltaic models. *Energy Sci. Eng.* **10**, 2035–2064. 10.1002/ese3.1115 (2022).

[46] Hassan, A. Y. et al. Evaluation of weighted mean of vectors algorithm for identification of solar cell parameters. *Processes* **10**, 1072. 10.3390/pr10061072 (2022).

[47] Premkumar, M. et al. Identification of solar photovoltaic model parameters using an improved gradient-based optimization algorithm with chaotic drifts. *IEEE Access* **9**, 62347–62379. 10.1109/access.2021.3073821 (2021).

[48] Malki, A., Mohamed, A. A., Rashwan, Y. I., El-Sehiemy, R. A. & Elhosseini, M. A. Parameter identification of photovoltaic cell model using modified elephant herding optimization-based algorithms. *Appl. Sci.* **11**, 11929. 10.3390/app112411929 (2021).

[49] Abdelghany, R. Y. et al. Development of an improved bonobo optimizer and its application for solar cell parameter estimation. *Sustainability* **13**, 3863. 10.3390/su13073863 (2021).

[50] Ismaeel, A. A. K., Houssein, E. H., Oliva, D. & Said, M. Gradient-based optimizer for parameter extraction in photovoltaic models. *IEEE Access* **9**, 13403–13416. 10.1109/access.2021.3052153 (2021).

[51] Chin, V. J. & Salam, Z. Coyote optimization algorithm for the parameter extraction of photovoltaic cells. *Solar Energy* **194**, 656–670. 10.1016/j.solener.2019.10.093 (2019).

[52] Ayyarao, T. L. V. & Kumar, P. P. Parameter estimation of solar pv models with a new proposed war strategy optimization algorithm. *Int. J. Energy Res.* **46**, 7215–7238. 10.1002/er.7629 (2022).

[53] Wolpert, D. & Macready, W. No free lunch theorems for optimization. *IEEE Trans. Evolut. Comput.* **1**, 67–82. 10.1109/4235.585893 (1997).

[54] Zhong, C. et al. Starfish optimization algorithm (SFOA): A bio-inspired metaheuristic algorithm for global optimization compared with 100 optimizers. *Neural Comput. Appl.* **37**, 3641–3683. 10.1007/s00521-024-10694-1 (2024).

[55] Lian, J. et al. The educational competition optimizer. *Int. J. Syst. Sci.* **55**, 3185–3222. 10.1080/00207721.2024.2367079 (2024).

[56] Amiri, M. H., Mehrabi Hashjin, N., Montazeri, M., Mirjalili, S. & Khodadadi, N. Hippopotamus optimization algorithm: A novel nature-inspired optimization algorithm. *Sci. Rep.* **14**, 10.1038/s41598-024-54910-3 (2024). 10.1038/s41598-024-54910-3 PMC10904400 38424229

[57] Dehghani, M. & Trojovský, P. Osprey optimization algorithm: A new bio-inspired metaheuristic algorithm for solving engineering optimization problems. *Front. Mech. Eng. ***8**, 10.3389/fmech.2022.1126450 (2023).

[58] Trojovská, E., Dehghani, M. & Trojovský, P. Zebra optimization algorithm: A new bio-inspired optimization algorithm for solving optimization algorithm. *IEEE Access* **10**, 49445–49473. 10.1109/access.2022.3172789 (2022).

